# Structure Elucidation of Fucan Sulfate from Sea Cucumber *Holothuria fuscopunctata* through a Bottom-Up Strategy and the Antioxidant Activity Analysis

**DOI:** 10.3390/ijms23094488

**Published:** 2022-04-19

**Authors:** Li Gao, Chen Xu, Xuelin Tao, Zhichuang Zuo, Zimo Ning, Linghui Wang, Na Gao, Jinhua Zhao

**Affiliations:** 1 School of Pharmaceutical Sciences, South-Central University for Nationalities, Wuhan 430074, China; gl15272975735@163.com (L.G.); 15171981226@163.com (C.X.); txl6361@163.com (X.T.); 18271682301@163.com (Z.Z.); zimo_ning1997@163.com (Z.N.); wlh705127994@163.com (L.W.); 2 National Demonstration Center for Experimental Ethnopharmacology Education, South-Central University for Nationalities, Wuhan 430074, China

**Keywords:** fucan sulfate, mild acid hydrolysis, oligosaccharides, structure, antioxidant activity

## Abstract

Fucan sulfate I (FSI) from the sea cucumber *Holothuria fuscopunctata* was purified and its structure was clarified based on a bottom-up strategy. The unambiguous structures of a series of oligosaccharides including disaccharides, trisaccharides, and tetrasaccharides, which were released from mild acid hydrolysis of FSI, were identified by one-dimensional (1D)/two-dimensional (2D) nuclear magnetic resonance (NMR) and mass spectrometry (MS) analysis. All the glycosidic bonds in these oligosaccharides were presented as α1,3 linkages confirmed by correlated signals from their ^1^H-^1^H ROESY and ^1^H-^13^C HMBC spectra. The structural sequence of these oligosaccharides formed by Fuc_2S4S_, Fuc_2S_, and non-sulfated ones (Fuc_0S_), along with the general structural information of FSI, indicated that the structure of FSI could be elucidated as: [-L-Fuc_2S4S_-α1,3-L-Fuc_(2S)_-α1,3-L-Fuc_2S_-α1,3-L-Fuc_0S_-α1,3-1-]*_n_*. Moreover, the L-Fuc_0S-_α1,3-L-Fuc_2S4S_ linkage in FSI was susceptible to be cleaved by mild acid hydrolysis. The antioxidant activity assays in vitro showed that FSI and the depolymerized product (dFSI′) had potent activities for superoxide radical scavenging activity with IC_50_ of 65.71 and 83.72 μg/mL, respectively, while there was no scavenging effect on DPPH, hydroxyl and ABTS radicals.

## 1. Introduction

Sea cucumber has been recognized as a type of high-value marine food in many parts of Asia. It contains nutritional and functional components, such as proteins, collagen, saponins, and sulfated polysaccharides, mainly including fucan sulfate (FS) and fucosylated chondroitin sulfate, which possess multiple biological activities including antioxidant, antithrombotic, anticancer, immunoregulatory activity, anti-inflammatory, antibacterial, antidiabetic, antiobesity and antiangiogenic activities [1,2,3].

FS is a natural sulfated polysaccharide mainly composed of sulfated fucose (Fuc) residues and originally extracted from the extracellular matrix of brown seaweeds, which is denominated as fucoidan specifically [4]. In general, fucoidan from brown algae is usually known as a complex heteropolysaccharide, and high variability has been observed in terms of glycosidic bond types, sulfation substitution types, and monosaccharides [5]. Furthermore, it usually contains other monosaccharides such as glucose, galactose, and mannose [6]. Due to the structural complexity of fucoidan, elucidation of its exact structure and the correlation between structure and biological function is hindered. Interestingly, FS with a regular structure has been found in sea cucumbers and egg jellies of sea urchins [7]. Compared with fucoidans extracted from brown seaweed, most FS from sea cucumber generally possess a simple and linear structure, which facilitates study of its structure–activity relationship. Up to now, in addition to the highly regular homogeneous FS consisting of uniform sulfated fucose residues and glycosidic bonds [8], it was also found that the primary structure of FS from different sea cucumbers is composed of a tetrafucose repeating unit linked by α1,3 and/or α1,4 glycosidic linkages with different sulfation patterns, such as FS from *Ludwigothurea grisea* [9], *Isostichopus badionotus* [10], *Acaudina molpadioides* [11,12], *Holothuria tubulosa* [13] and *Thelenota ananas* [14]. Interestingly, the basic structure of fucan sulfate I (FSI) in *Holothuria fuscopunctata* was described as a tetrasaccharide repeating unit that seems to be connected by unique alternating α1,3 and α1,4 glycosidic bonds [15] The structure was stated as: [-L-Fuc_2S4S_-α1,4-L-Fuc-α1,3-L-Fuc_2S_-α1,4-L-Fuc-α1,3-]*_n_*. However, a previous report about the structure of FSI was based on the monosaccharide composition and one-dimensional (1D)/two-dimensional (2D) nuclear magnetic resonance (NMR) assignments of the low-molecular-weight product depolymerized by the free-radical cleavage method. In fact, the signals in the NMR spectra were overlapped and complex due to the various sulfation types, especially the H-3 and H-4 signals of Fuc_0S_, which were difficult to distinguish. Consequently, it is necessary to clarify the exact glycosidic bond types and the structure sequence in natural FSI through structural analysis of purified oligosaccharides. Obviously, an appropriate glycosidic bond cleavage method, further purification and precise structure elucidation of the oligosaccharide are of great advantage for the structural construction of the native FSI.

Methods for cleavage of the glycosidic bonds have been applied in the structural analysis of FS from marine invertebrates. Generally, enzymatic hydrolysis, peroxide depolymerization, and acid hydrolysis have been described for the depolymerization of FS [16,17,18]. However, the application of fucanases is limited to specifically hydrolyzing the structural sequence, with definite glycosidic linkages formed by sulfated fucose. For the widely used peroxide depolymerization method, the cleavage position of the polysaccharide chain may not be limited to the glycosidic bond, and this process would cause over oxidation of sugar ring carbon [18]. For example, in the case of FS connected by 1,4 glycosidic bonds, the breakdown of the C2–C3 bond in the sugar ring with the 2,3 ortho-dihydroxyl group may be observed. In addition, the oxidative cleavage of the reducing end residue may lead to the presence of complex terminal structures in the depolymerized products, which would hinder the purification and structure identification of the resulting fragments. Acid hydrolysis is considered as a common method to depolymerize FS due to the sensitivity of deoxyhexose glycosidic bonds to acid [17,19,20], and does not cause the breakdown of the C–C bond in the sugar ring. This method has been used in the structure analysis of FS from the sea urchin *Lytechinus variegatus*, the sea cucumber *Isostichopus badionotus* and *Pearsonothuria graeffei* [10,21,22]. Therefore, to confirm the exact structure of FSI from the sea cucumber *Holothuria fuscopunctata*, the mild acid hydrolysis method was employed in this study to obtain low-molecular-weight derivatives and released oligosaccharides. These oligosaccharide fragments were purified by gel permeation chromatography (GPC), and their structures were characterized by 1D/2D NMR and electrospray ionization mass spectrometry (ESI-MS) techniques. Through a bottom-up strategy, the structure of FSI was clearly assigned, thus confirming the connection mode of glycosidic bonds and repeating unit.

Moreover, fucan sulfate is known to be one of the most widespread sulfated polysaccharides distributed in marine organisms and it has been reported to possess diverse biological effects, suggesting its potential value in food and medical applications [4,23]. Given that the anticoagulant activity of FSI has been reported in our previous paper [15], along with abundant studies showing that FS possesses antioxidant activity, here the antioxidant activity of FSI and dFSI′ (dFSI′ is the low-molecular-weight derivative with Molecular weight (Mw) about 4.6 kDa, prepared through peroxide depolymerization) was evaluated in vitro by four free-radical generation systems.

## 2. Results and Discussion

### 2.1. Optimization of Reaction Temperature and Preparation of Mild Acid Hydrolyzed Product (dFSI)

**Figure 1:**
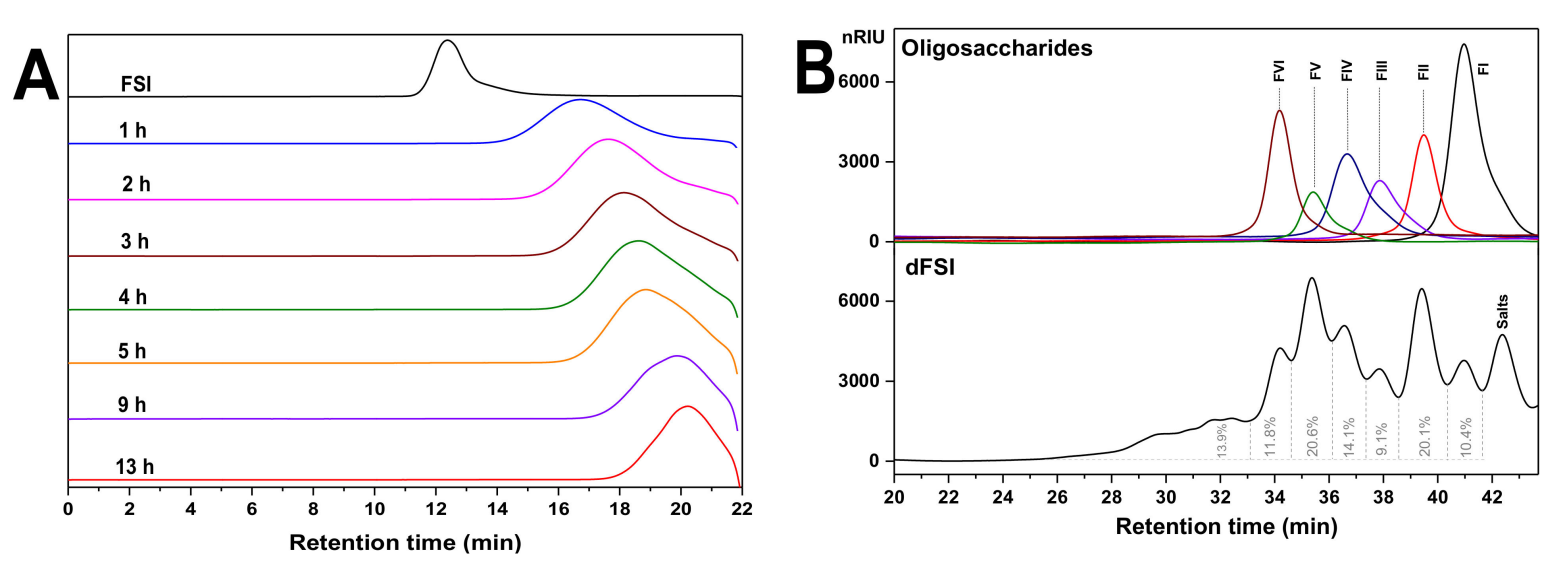
High-performance gel permeation chromatography (HPGPC) profiles of the products released from mild acid hydrolysis of fucan sulfate I (FSI). (**A**) HPGPC of the hydrolyzed products at different times at 80 °C determined on a Shodex OHpak SB-804 HQ column (8.0 mm × 300 mm) eluted with 0.1 M NaCl solution at a flow rate of 0.5 mL/min detected by refractive index detector (RID), respectively. (**B**) HPGPC profiles of mild acid hydrolyzed product (dFSI) and its oligosaccharides analyzed on a Superdex peptide 10/300 GL column eluted with 0.2 M NaCl solution at a flow rate of 0.4 mL/min detected by RID.

Mild acid hydrolysis is the most commonly used method for the cleavage of glycosidic bonds of complex polysaccharides [19]. Under 80 °C, the results showed that the retention time (Rt) of FSI was detected to change from 12 min to 17.628 min on a Shodex OHpak SB-804 HQ column after 2 h, while under 100 °C, after 2 h, Rt of the hydrolysate was detected at 20.982 min (Figure S1). Obviously, the mild hydrolysis rate of FSI under 100 °C was faster than under 80 °C and was not easy to control. Thus, FSI was depolymerized at 80 °C and dFSI was obtained. High-performance gel permeation chromatography (HPGPC) showed that its molecular weight was significantly reduced as the time course prolonged (Figure 1A). After reaching the appropriate molecular weight, 6 M NaOH was added to terminate the reaction and the hydrolysates were desalted on a Sephadex G-25 column, then freeze-dried to produce 1.4 g dFSI with a yield of 78%.

### 2.2. Purification of Size-Homogeneous Oligosaccharides from dFSI

**Figure 2:**
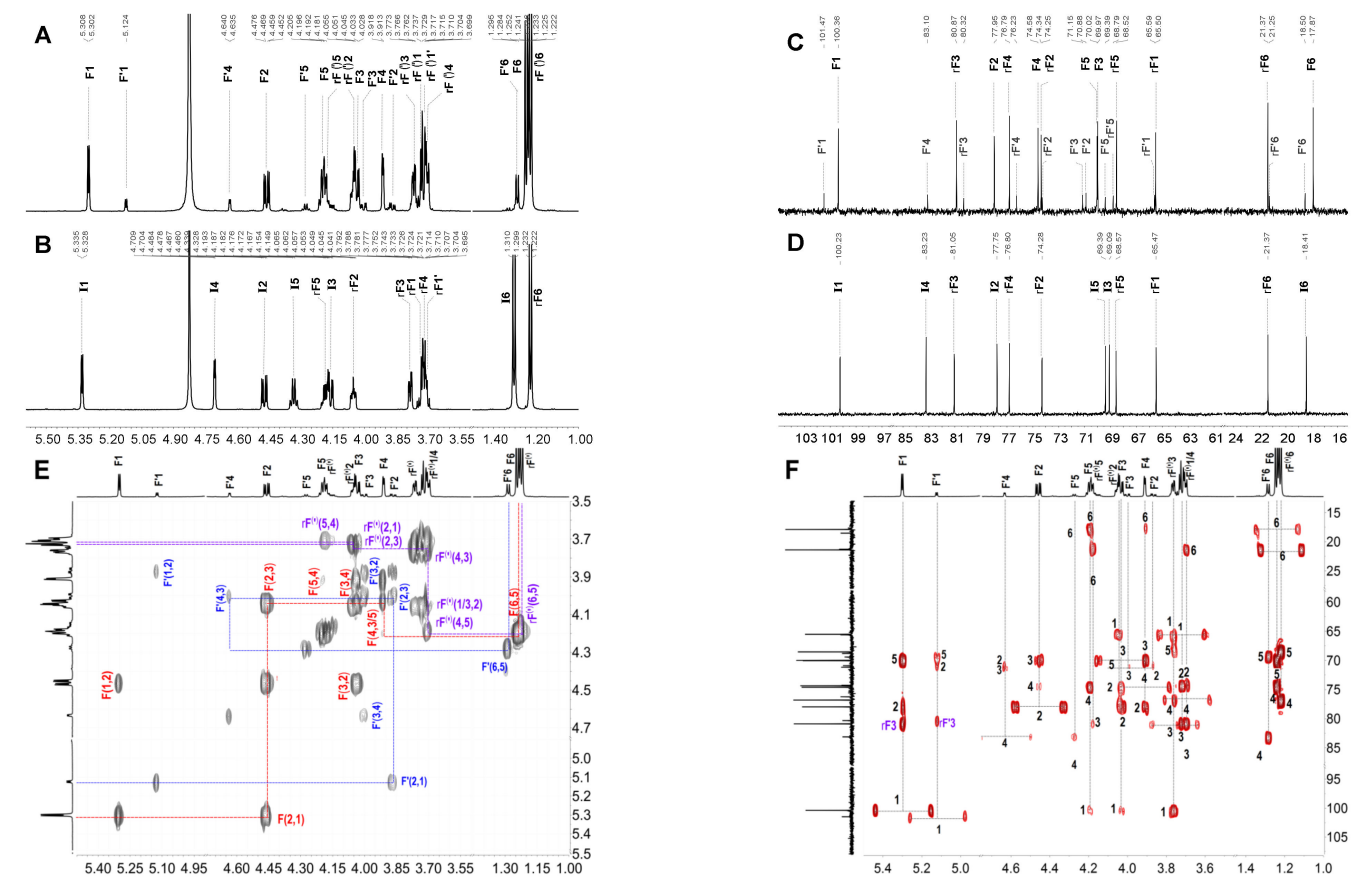

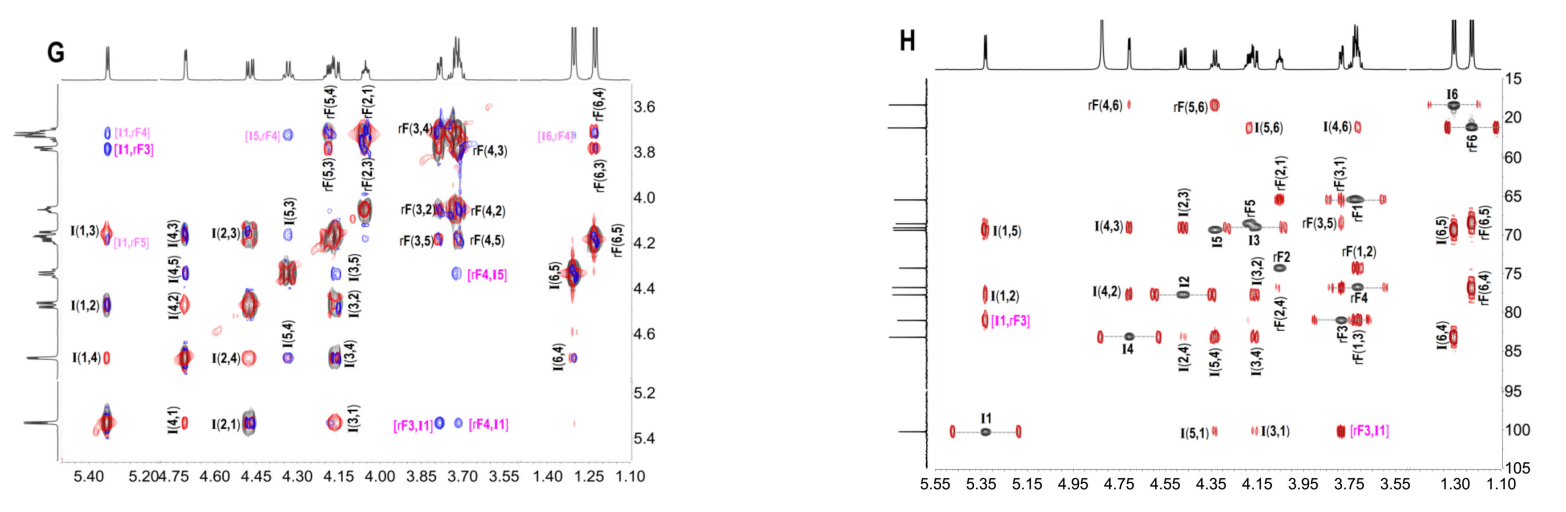
The ^1^H (**A**,**B**) and ^13^C (**C**,**D**) spectra of FI and FII, correlation spectroscopy(^1^H-^1^H COSY) (**E**) and heteronuclear multiple bond correlation (^1^H-^13^C HMBC) (**F**) of FI; the overlapped spectra (**G**) of ^1^H-^1^H COSY (gray), total correlation spectroscopy (^1^H-^1^H TOCSY)(red) and rotating frame Overhauser effect spectroscopy (^1^H-^1^H ROESY) (blue) of FII; and the overlapped spectra (**H**) of heteronuclear single quantum coherence (^1^H- ^13^C HSQC) (gray) and ^1^H- ^13^C HMBC (red).

**Figure 3:**
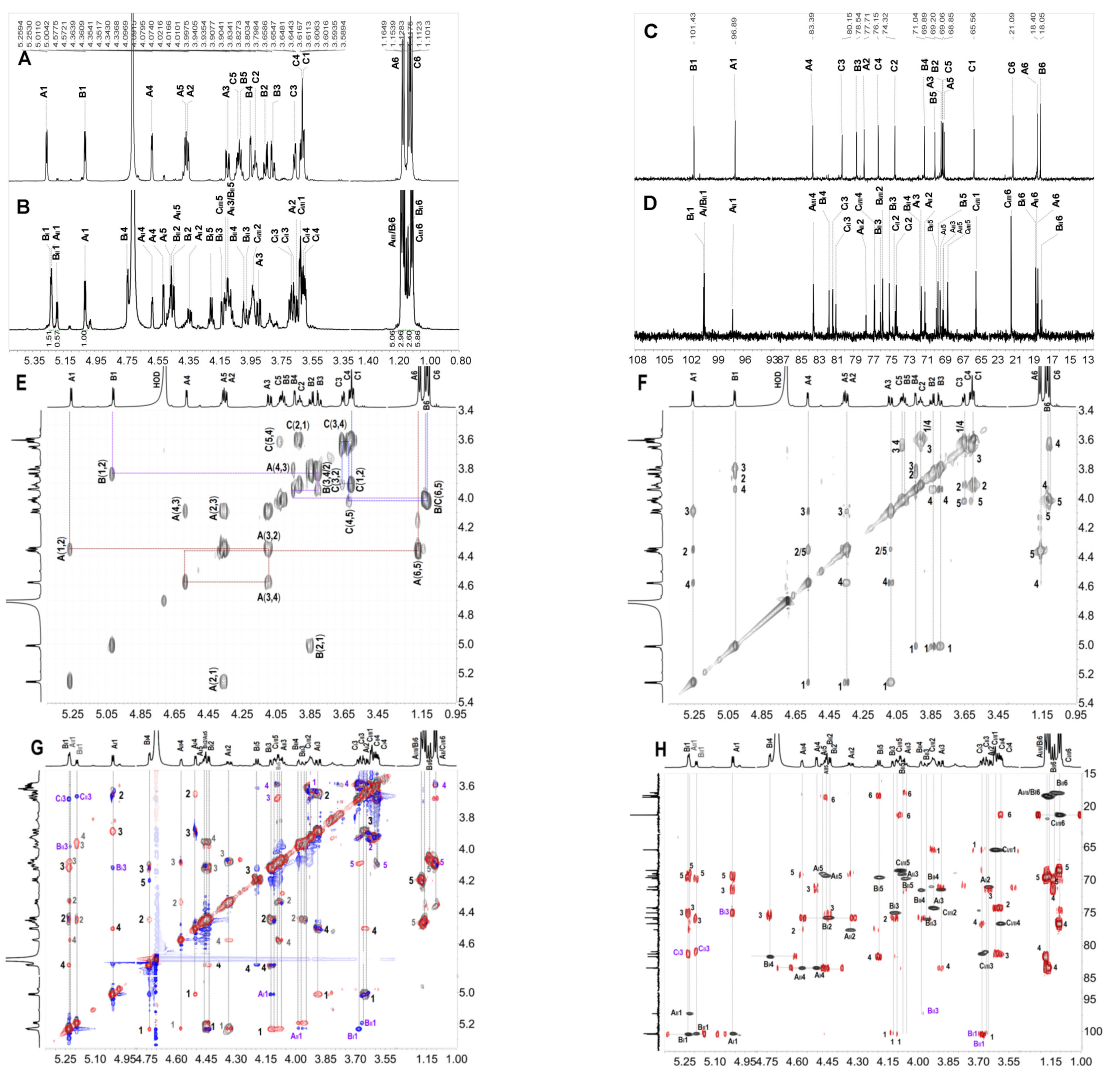
The ^1^H (**A**,**B**) and ^13^C (**C**,**D**) spectra of FIII and FIV, ^1^H–^1^H COSY (**E**) and ROESY (**F**) of FIII; the overlapped ^1^H–^1^H COSY (gray), TOCSY (red) and ROESY (blue) spectra (**G**) of FIV; and the overlapped ^1^H-^13^C HSQC (gray) and ^1^H-^13^C HMBC (red) spectra (**H**).

**Figure 4:**
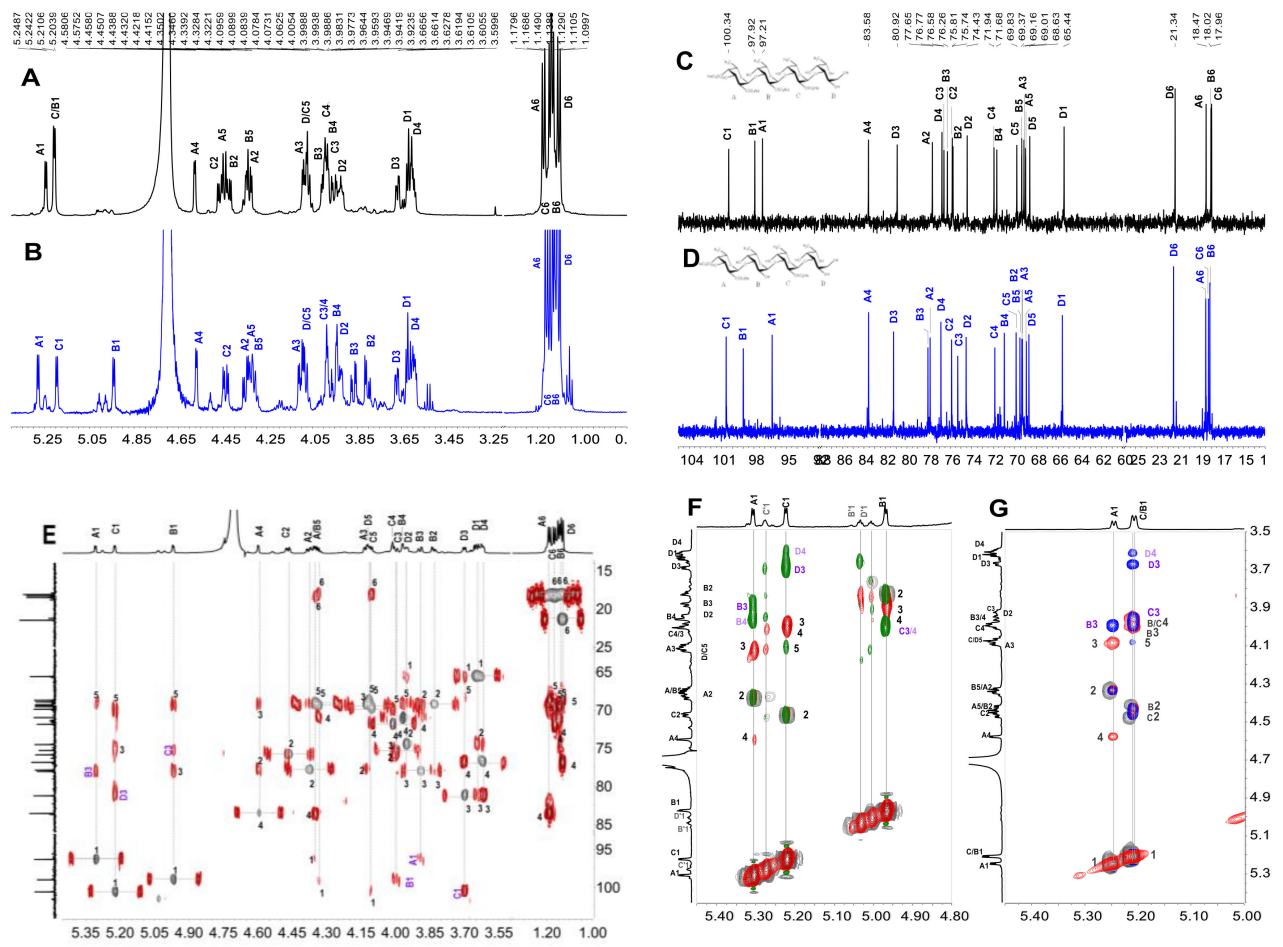
The ^1^H (**A**,**B**) and ^13^C (**C**,**D**) spectra of FVI and FV; the overlapped ^1^H-^13^C HSQC (gray) and ^1^H-^13^C HMBC (red) spectra (**E**) of FV, partial ^1^H-^1^H COSY (gray), ^1^H-^1^H TOCSY (red) and ^1^H-^1^H ROESY (green in (**F**) and blue in (**G**)); and the overlapped spectra of FV (**F**) and FVI (**G**).

**Figure 5:**
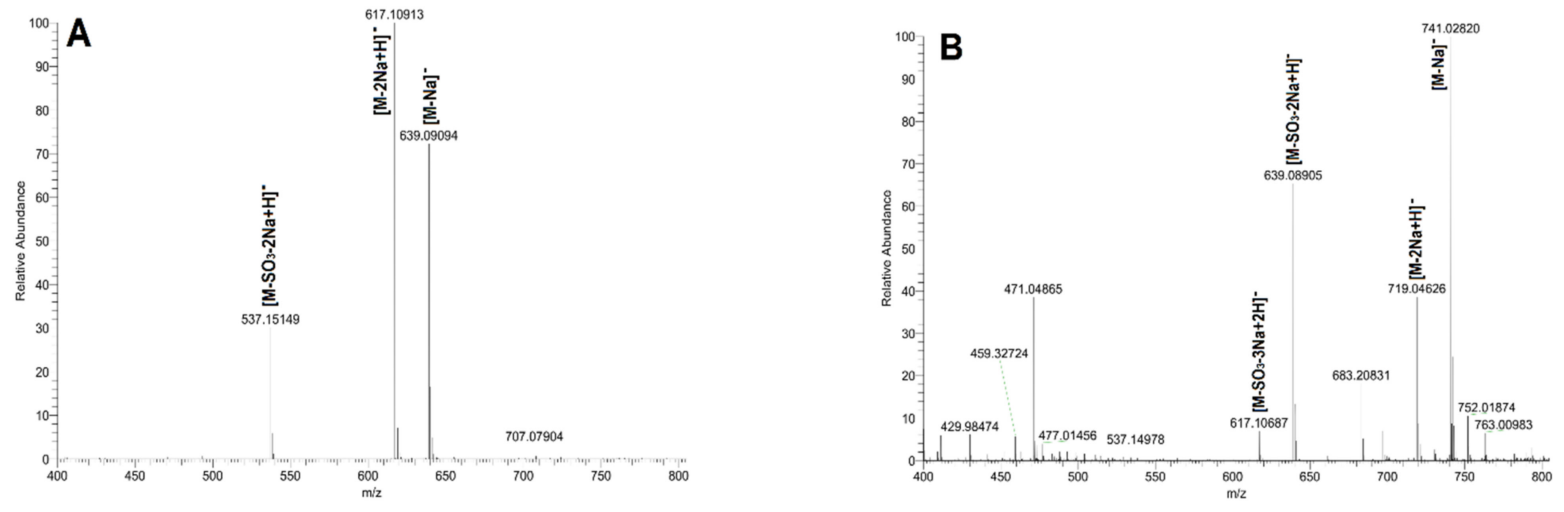

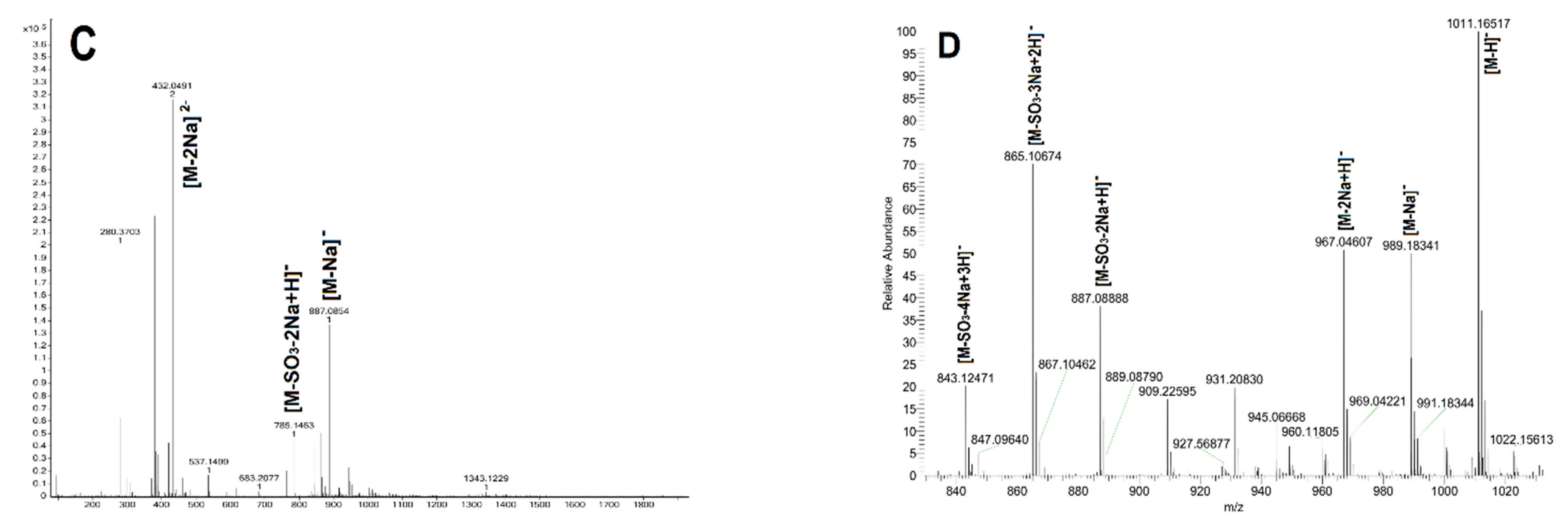
The mass spectrometry (MS) spectra of oligosaccharides FIII (**A**), FIV (**B**), FV (**C**) and FVI (**D**).

Native FSI showed a ^1^H NMR spectrum with broad signals due to its high Mw (Figure S2) [15], thus hindering the detailed structural determination. For further detailed structural analysis, optimized conditions (10 mM trifluoroacetic acid, 80 °C and 13 h) were used for large-scale preparation of oligosaccharides. The HPGPC profiles of dFSI shown in Figure 1B reveal that it was composed of at least six fractions with different Mw. Thus, it was fractionated by GPC on a Bio-Gel P-6 or P-4 column. According to the elution profile determined by the phenol-sulfuric acid method, six major fractions were observed, which is in accordance with the HPGPC results. Then, each fraction was further analyzed by HPGPC and pooled for further purification. Finally, six fractions (FI, FII, FIII, FIV, FV, and FVI) were obtained and their HPGPC analysis showed single symmetric peaks corresponding to those peaks in dFSI with different Rt, indicating that FI–FVI are all size-homogeneous oligosaccharides. Then, FI–FVI were subjected to detailed structural analysis by 1D/2D NMR (Figure 2, Figure 3 and Figure 4, Table 1, Table 2 and Table 3) and mass spectrometry (MS) (Figure 5, Table 4), and these data could be used to deduce the structure of native FSI through a bottom-up strategy.

### 2.3. Structural Determination of Oligosaccharides by NMR

**Scheme 1:**
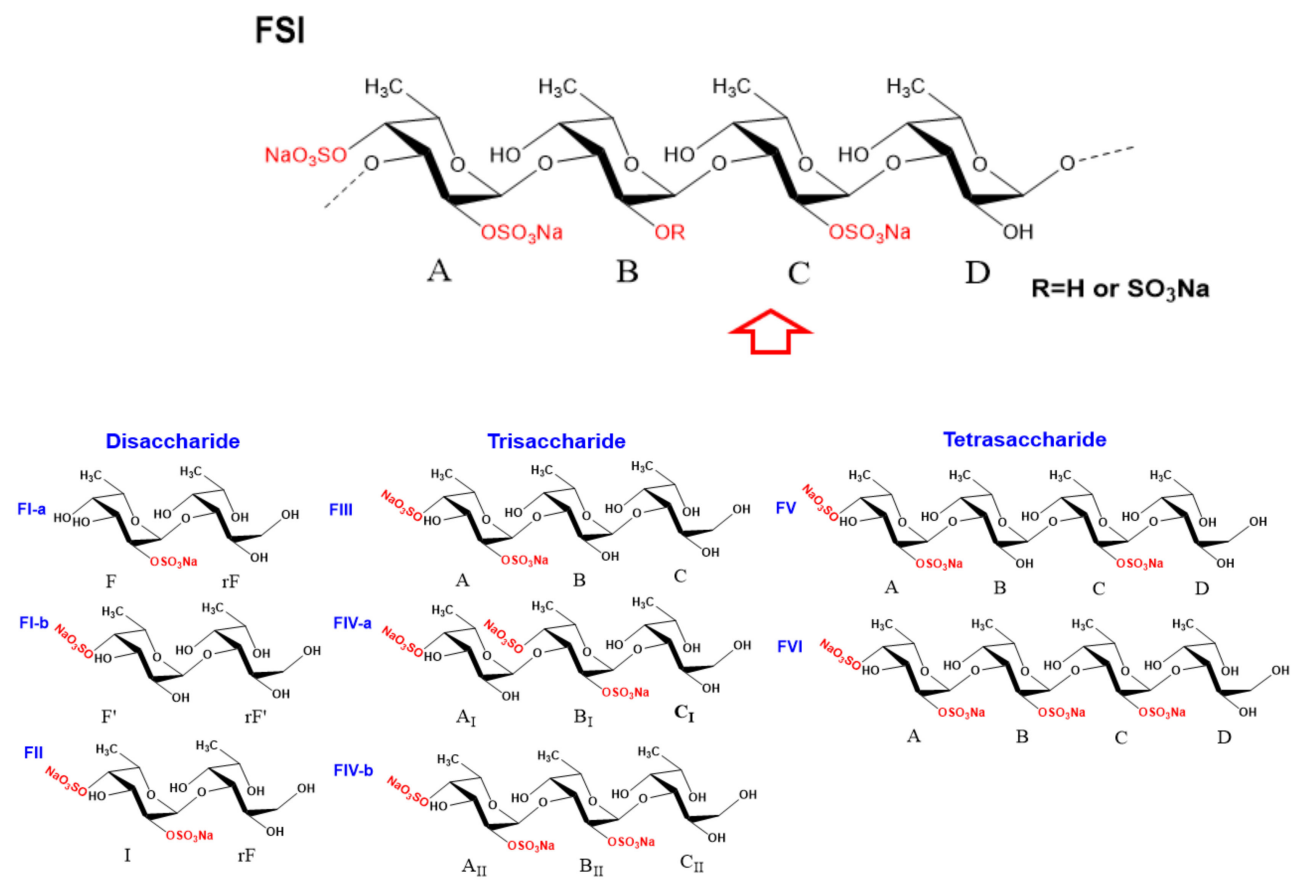
The basic structure of FSI was confirmed with bottom-up strategy. The letters below the structure of each oligosaccharide are used as labels for assignments in the NMR spectra, respectively.

With the availability of high-resolution NMR technology, the fine structure of the complex sulfated polysaccharides from marine organisms and various oligosaccharides can be readily elucidated. In the region of anomeric protons in the ^1^H NMR (Figure 2A) spectrum of FI, a set of distinct signals and a set of weak signals were observed at 5.305 and 5.124 ppm, respectively, which could be assigned to the α-anomeric protons of fucose residues. From these α-anomeric protons, two distinct spin–spin coupling systems were observed in the correlation spectroscopy (^1^H-^1^H COSY) (Figure 2E) of FI, implying that it was a disaccharide mixture (FI-a and FI-b). Strong signals around 1.1–1.3 ppm in the ^1^H NMR spectrum of FI could be assigned to the typical signals of methyl protons in L-fucose residue. Additionally, the ^1^H and ^13^C chemical shifts were fully assigned unambiguously according to the ^1^H-^1^H COSY, total correlation spectroscopy (^1^H-^1^H TOCSY), and heteronuclear single quantum coherence (^1^H-^13^C HSQC) spectra (Figure 2E, Figure S3A,C and Table 1). The H-2 of the fucose residue (F) located at the non-reducing end of FI-a was observed at 4.463 ppm, which shifted downfield ~0.4 ppm compared with the non-sulfated fucose, indicating this residue F was sulfated at the C-2 position. Similarly, the residue at the non-reducing end (F′) in FI-b should be sulfated at the C-4, which was confirmed by the H-4 at 4.638 ppm. The sulfated types of F in FI-a and F′ in FI-b could further be confirmed by the downfield signal of the corresponding carbons (C-2 of F at 77.96 ppm and C-4 of F′ at 83.11 ppm). The H-1-H-6 and C-1-C-6 assignments (Figure 2C, Table 1) of the reducing end residue in FI-a (rF) and FI-b (rF′) showed that they were non-sulfated fucose, and they were reduced to the corresponding fucitol. The H-1 of rF and rF′ were observed at about 3.7 ppm and the C-1 were at 65.51 and 65.60 ppm, respectively. Correlations were found between H-1 (5.305 ppm) of F and H-3 (3.769 ppm) of rF in the rotating frame Overhauser effect spectroscopy (^1^H-^1^H ROESY) (Figure S3B), confirming that the glycosidic bond between residue F and rF in FI-a was 1,3; this was verified by the correlation of H-1 (5.305 ppm) of F and C-3 (80.88 ppm) of rF in the heteronuclear multiple bond correlation (^1^H-^13^C HMBC) (Figure 2F). Similarly, the glycosidic linkage between F′ and rF′ in FI-b was determined as 1,3 linkages. The residues F in FI-a and F′ present in FI-b were confirmed to be in α-configuration, since the coupling constants were *J*_1,2_ = 4.05 and *J*_1,2_ = 4.20, respectively. These results allowed us to conclude that FI was composed of two disaccharides with structure sequences of L*-*Fuc_2S_*-*α1,3*-*L*-*Fuc*-*ol (FI-a) and L*-*Fuc_4S_*-*α1,3*-*L*-*Fuc*-*ol (FI-b) (Scheme 1), respectively. The integral ratios of the α-anomeric protons at 5.305 and 5.124 ppm could show the content of these two disaccharides were 83 and 17%. These results were consistent with those of the assignment of the ESI-Q-TOF MS data (Figure S3D, Table 4).

Similarly, FII showed clear signals in its ^1^H and ^13^C NMR spectra (Figure 2B,D). Starting from the unambiguous signals at 5.332 ppm, one spin system was found in the ^1^H–^1^H COSY spectrum (Figure S4A), indicating that it was a fucose unit. Another spin–spin system from signals at 3.71 and 3.69 ppm was observed. According to the integration of proton signals in the ^1^H NMR spectrum and the total number of carbons in the ^13^C NMR (Figure 2B,D), it could be deduced to be a disaccharide. The ^1^H and ^13^C chemical shifts of FII can be readily assigned and are shown in Table 1. Compared with the non-sulfated fucose residues, the obvious downfield resonances of the H-2 signal (δ 4.472 ppm) and H-4 signal (δ 4.706 ppm) of the non-reducing end residue (I) indicated this fucose was sulfated at both C-2 and C-4 positions, while the reducing end was L-Fuc-ol. The glycosidic bond was clarified as 1,3 according to the correlation peaks in ^1^H-^1^H ROESY and ^1^H-^13^C HMBC (Figure 2G,H). Additionally, the molecular formula was determined to be C_12_H_22_Na_2_O_15_S_2_ by negative ESI-MS peaks at *m*/*z* = 493.0324 assigned as [M-Na]^−^ (calculated as 493.0298), 471.0502 assigned as [M-2Na+H]^-^ (calculated as 471.0478), and 391.0933 assigned as [M-SO_3_-2Na+H]^-^ (calculated as 391.0910) (Figure S4B). In summary, its structure can be stated as L-Fuc_2S4S_-α1,3-L-Fuc-ol (Scheme 1).

Furthermore, comparing the ^1^H and ^13^C NMR spectra (Figure 3A,C) of FIII with the spectra of FII, FII**I** showed more signals with clear resolution. The presence of three spin–spin systems in ^1^H–^1^H COSY (Figure 3E) suggested FIII should be a trisaccharide and the three fucose residues were listed as A, B and C, respectively. The chemical shift assignments of other proton and carbon signals of FIII could be assigned completed by analyzing ^1^H–^1^H TOCSY, ^13^C and ^1^H–^13^C HSQC (Figure S5A,B); these are listed in Table 2. Since the H-2 (4.348 ppm) and H-4 (4.575 ppm) signals of residue A apparently shifted to downfield, residue A was deduced as 2,4-O-di-sulfated fucose residue, which could also be confirmed by the chemical shift of C-2 (77.71 ppm) and C-4 (83.40 ppm). Similarly, residues B and C could be clarified as non-sulfated fucose and residue C was presented as its fucitol form. The glycosidic linkage could be revealed by the assignments of the correlation peaks in the ^1^H-^1^H ROESY (Figure 3F) and ^1^H-^13^C HMBC (Figure S5C). In particular, the correlation from the H-1 of residue A to the H-3 of residue B, and the correlation from the H-1 of residue B to the H-3 of residue C were observed, suggesting residues A, B, and C were all connected with 1,3 glycosidic bonds. The small coupling constants of residue A (*J*_1,2_ = 3.84) and residue B (*J*_1,2_ = 4.08) indicate the α-anomeric sugar configuration. Taken together, the structure sequence of FIII was demonstrated as L-Fuc_2S4S_-α1,3-L-Fuc-α1,3-L-Fuc-ol (Scheme 1).

In the same way, FIV should be a mixture of two trisaccharides (FIV-a and FIV-b). They shared the same glycosidic bond type with FIII and were clarified as α1,3 (Figure 3 and Figure S6, Scheme 1). The most distinct structural characteristic of FIV was the sulfated types of the fucose residues—in other words, the structure sequence. Unlike the FIII, FIV has three positions substituted by sulfate groups. For FIV-a, the downfield H-4 (4.504 ppm) of residue A_I_, and H-2 (4.438 ppm) and H-4 (4.736 ppm) of residue B_I_ (Figure 3B,G and Table 2) indicated that the position of C-4 of residue A_I_ was substituted by the sulfate group, and the positions of C-2 and C-4 of residue B_I_ were both sulfated. Likely, the residue A_II_ of FIV-b was clarified as Fuc_2S4S_, and residue B_II_ was Fuc_2S_. Combining the above information, the structure of FIV-a and FIV-b could be unambiguously inferred as L-Fuc_4S_-α1,3-L-Fuc_2S4S_-α1,3-L-Fuc-ol and L-Fuc_2S4S_-α1,3-L-Fuc_2S_-α1,3-L-Fuc-ol (Scheme 1), respectively. Since FIV-a and FIV-b were trisaccharides with three sulfate groups, and FIII was a trisaccharide with two sulfate groups, they showed different retention times in their HPGPC profiles. The chemical formula (C_18_H_31_Na_3_O_22_S_3_) was determined based on the ESI-MS data (Figure 5, Table 4).

By analyzing the 1D/2D NMR spectra of FV (Figure 4B,D–F and Figure S7), we found that it consists of four tetrasaccharides (FV-a, FV-b, FV-c, and FV-d) linked by an α1,3 bond, and they are mainly different in the sulfated types of the fucose residue in the structure sequence. The structure of the main tetrasaccharide (FV-a) was identified as L-Fuc_2S4S_-α1,3-L-Fuc-α1,3-L-Fuc_2S_-α1,3-L-Fuc-ol (Scheme 1), and the structures of minor components (FV-b, FV-c, FV-d) were confirmed as L-Fuc_4S_-α1,3-L-Fuc_2S4S_-α1,3-L-Fuc-α1,3-L-Fuc-ol, L-Fuc_2S_-α1,3-L-Fuc-α1,3-L-Fuc_2S4S_-α1,3-L-Fuc-ol, and L-Fuc_4S_-α1,3-L-Fuc_4S_-α1,3-L-Fuc_2S_-α1,3-L-Fuc-ol, respectively (Figure S7).

FVI was similar to FV-a, and it was also a tetrasaccharide (Figure 4A,C,G and Figure S8). However, the distinct difference is that the downfield chemical shifts of H-2 (4.427 ppm) of residue B (Table 3), compared with non-sulfated fucose residues, indicated that residue B was sulfated at the C-2. In general, the structure of FVI was stated as L-Fuc_2S4S_-α1,3-L-Fuc_2S_-α1,3-L-Fuc_2S_-α1,3-L-Fuc-ol (Scheme 1).

### 2.4. Structural Characteristics of Fucan Sulfate I (FSI) through a “Bottom-Up” Strategy

**Figure 6:**
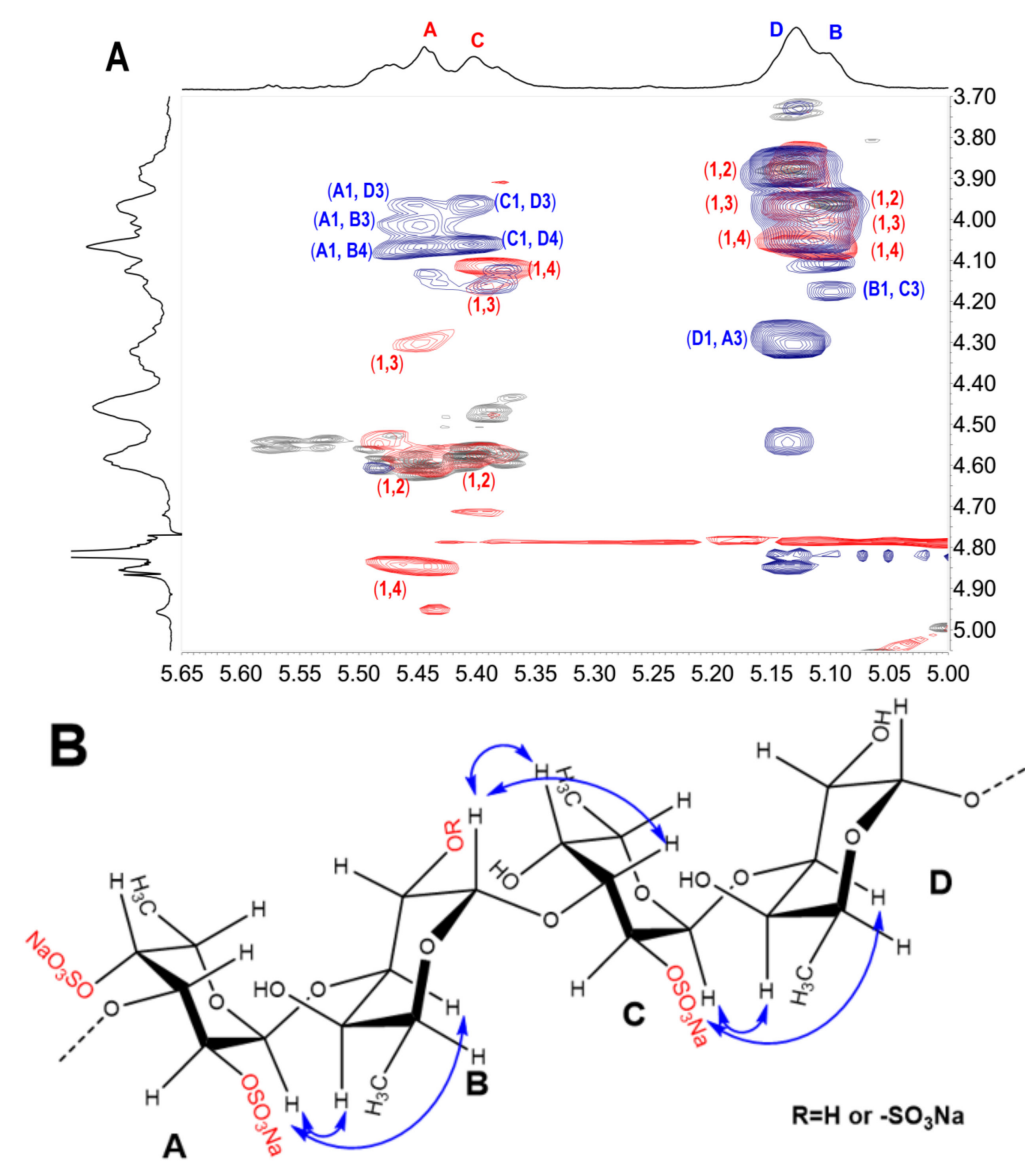
Partial overlapped spectra (**A**) of ^1^H-^1^H COSY (gray), ROESY (blue) and TOCSY (red) of depolymerized product (dFSI′) and possible cross-peaks from H-1 in ROESY marked by blue arrows (**B**). The capital letters A, B, C and D represent Fuc_2S4S_, Fuc_(2S)_, Fuc_2S_ and Fuc, respectively.

From the structural analysis of oligosaccharides, we find that the glycosidic bonds in the oligosaccharides from FSI were in the form of α1,3 connections (Scheme 1). This was different from the previous report in that the basic structural units of FSI isolated from *H. fuscopunctata* were connected by alternating α1,3 and α1,4 glycosidic bonds [15]. The previous report showed that the NMR signals of the native FSI and the peroxide depolymerization product were ambiguous, and were not easy to distinguish, which led to misjudgment of the exact sites of the glycosidic bond. In particular, the H-3 signals of residue B (Fuc) and residue D (Fuc) were all observed at about 4.07 ppm, and their H-4 signals exhibit nearly equal chemical shifts at 3.97 ppm. In the ^1^H–^1^H ROESY spectrum (Figure 6A), the correlated peaks between H-1 of residue A (Fuc_2S4S_) and H-3 or H-4 of residue B (Fuc_(2S)_), and the correlated peaks of H-1 of residue C (Fuc_2S_) and H-3 or H-4 of residue D, could be observed, indicating the presence of 1,3 or 1,4 glycosidic bonds between A (Fuc_2S4S_) and B (Fuc_(2S)_), and C (Fuc_2S_) and D (Fuc). In fact, inter-residue cross-peaks in the ROESY spectrum usually depended on proton–proton interatomic distances and related interatomic through-space connections (Figure 6B) [24,25]. The cross-peaks of anomeric proton H-1 and the H-4 of the subsequent unit could also be observed clearly in the ^1^H–^1^H ROESY spectra of oligosaccharides, including FI, FII, and FV.

Regarding the sulfated types of the Fuc residues, the substitutions are restricted at the C-2 and/or C-4 positions based on the structure of the trisaccharides and tetrasaccharides. The oligosaccharide FV was the main component in the acid hydrolysate of FSI (the bottom part of Figure 1B), suggesting the tetrasaccharide sequence L-Fuc_2S4S_-α1,3-L-Fuc-α1,3-L-Fuc_2S_-α1,3-L-Fuc should be the most prevalent building blocks in native FSI. The subsequent unit of Fuc_2S4S_ residue also could be present as Fuc_2S_, which is consistent with the structure sequence of FVI. Meanwhile, the heterogeneity in the structure of FSI was also observed from the structure sequence of oligosaccharides. For example, the 4-sulfated fucose residue linked to the Fuc_2S4S_ residue was shown in FIV-a, indicating the presence of a small amount of Fuc_4S_ residues in FSI. Comparison of the structure sequences of FIII and FIV-b, and FV and FVI, the Fuc_2S4S_ residue could be followed by non-sulfated fucose or 2-sulfated fucose (Fuc_2S_) residue. These results indicated that the main structure sequence of FSI should be constructed as tetrasaccharide repeating block [-L-Fu_2S4S_-α1,3-L-Fuc_(2S)_-α1,3-L-Fuc_2S_-α1,3-L-Fuc-α 1,3-], but with variability in sulfated patterns.

Regarding the possible process of mild acid hydrolysis of FSI, the non-reducing termini of trisaccharides and tetrasaccharides were mainly Fuc_2S4S_ residues, and the reducing ends were all fucitols of non-sulfated fucose (Fuc-ol), implying this depolymerized process should be a structure-dependent, selective glycosidic bond cleavage process. According to the literature, in the cases of sulfated fucan without non-sulfated fucose residues, the selective 2-desulfation occurs exclusively on the 2-sulfated fucose unit obligatorily linked to or preceded by a 4-sulfated fucose unit. Thus, the glycosidic linkage from this desulfated fucose unit was susceptible to acid and then could be preferentially cleaved [21,26]. Interestingly, for the presence of non-sulfated fucose unit in FSI, it was possible to ascertain that the glycosidic linkage from Fuc was preferentially cleaved. Combined with the ^1^H–^1^H ROSEY spectrum (Figure 6A) of the product obtained by free-radical depolymerization, the non-sulfated fucose residue (D) was clearly connected to 2,4-di-sulfated fucose unit (Fuc_2S4S_). The Fuc_2S4S_ residues at the non-reducing termini and the Fuc-ol located at the reducing end of those oligosaccharides strongly suggest that the α1,3 glycosidic linkages between Fuc and Fuc_2S4S_ were selectively cleaved. This preferred cleavage may also be because the 4-sulfate groups could promote a steric and/or electrostatic protection of the glycosidic linkage of the polysaccharide, which could be proved by the presence of Fuc_4S_ at the non-reducing end of some oligosaccharides (FI-b, FIV-a).

In summary, our previous report suggested that the basic structural unit of FSI was [-L-Fuc_2S4S_-α1,4-L-Fuc-α1,3-L-Fuc_2S_-α1,4-L-Fuc-α1,3-]*_n_*, which was deduced by analysis of the ^1^H–^1^H ROESY spectrum of the low-molecular-weight product obtained by peroxide depolymerization. In the present study, through comprehensive analysis of the chemical structure of oligosaccharide fragments and using a bottom-up strategy, native FSI was proven to be composed of the main tetrasaccharide repeating unit with a structure sequence of [-L-Fu_2S4S_-α1,3-L-Fuc_(2S)_-α1,3-L-Fuc_2S_-α1,3-L-Fuc-α1,3-].

### 2.5. Antioxidant Activities of FSI and Its Low-Molecular-Weight Derivative dFSI′

**Figure 7:**
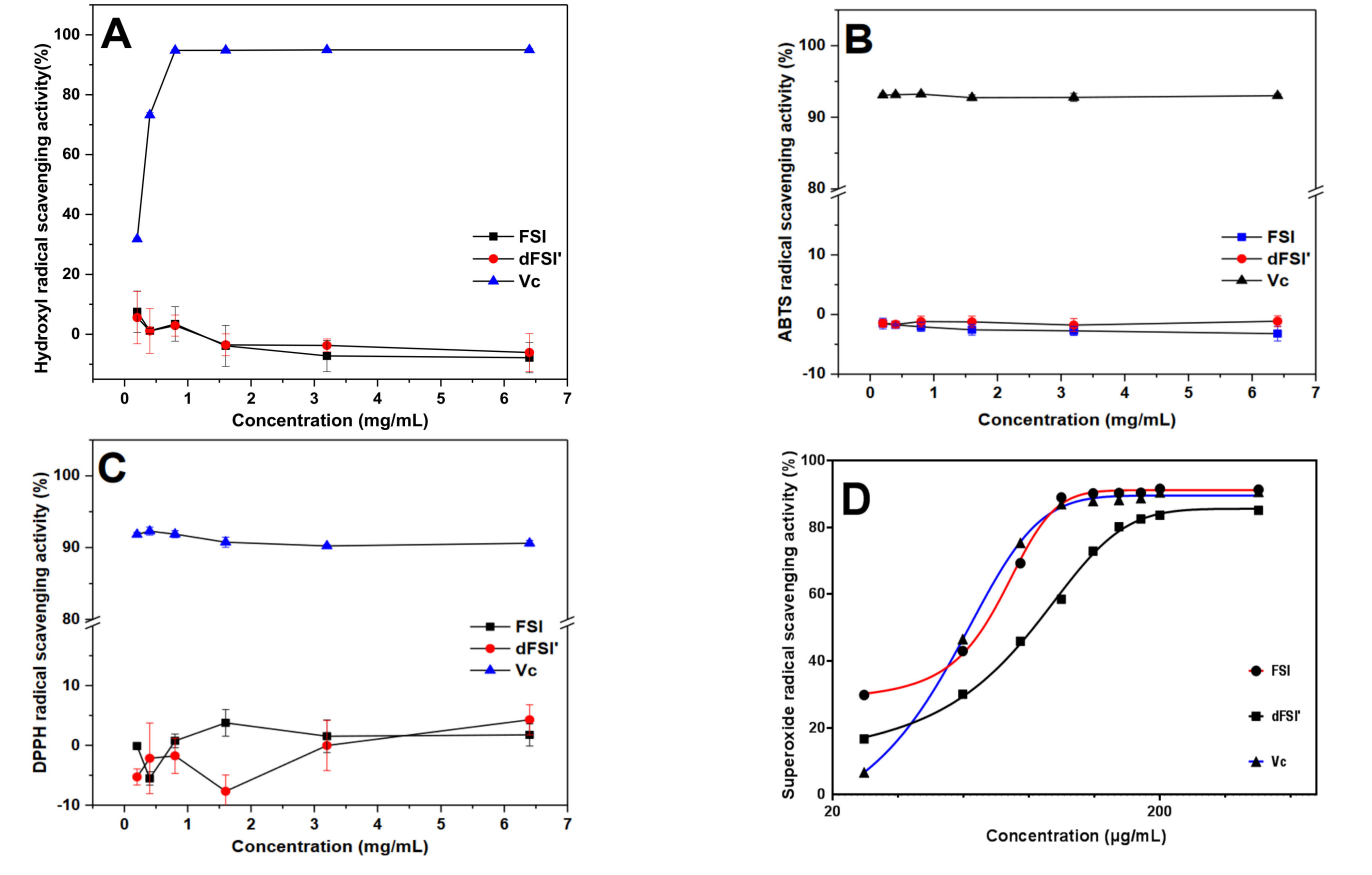
Scavenging effect of FSI and dFSI′ on hydroxyl radicals (**A**), ABTS radicals (**B**), DPPH radicals (**C**) and superoxide radicals (**D**), respectively. (*n* = 3).

It is widely known that reactive oxygen species (ROS) including superoxide anion (•O_2_^–^), hydroxyl radical (•OH) and hydrogen peroxide (H_2_O_2_) could cause significant damage to biomolecules such as DNA, lipids, and proteins in cells [6]. Sulfated polysaccharides, especially fucoidan and fucan, have been considered as antioxidant compounds by the food and pharmaceutical industries. Therefore, the antioxidant activities of FSI and its low-molecular-weight derivative dFSI′ (Mw 4.60 kDa) on hydroxyl radicals, (2,2′-azinobis-(3-ethyl-benzothiazolin-6-sulfonic acid)) diammonium salt (ABTS) radicals, 2,2-diphenyl-1-picrylhydrazyl radicals (DPPH•), and superoxide radicals were evaluated in vitro, respectively. The results of this study are shown in Figure 7 and indicate that fucan sulfate possesses radical scavenging activities corroborated by different methods in vitro. Compared with vitamin C at the concentration range of 0.2–6.4 mg/mL, FSI and dFSI′ exhibited negligible DPPH•, ABTS^•+^, and hydroxyl radicals scavenging activities. However, fucoidan from algae showed antioxidant activities in previous reports [27]. These inconsistent results may be due to the antioxidant activity of sulfated polysaccharides, depending on several structural characteristics, and the presence of proteins or polypeptides may also increase the radical scavenging effect. Interestingly, the scavenging ability of FSI and dFSI′ on superoxide radicals generated in the nicotinamide adenine dinucleotide-phenazine methosulfate (NADH-PMS) system was significant in a concentration-dependent fashion at a rather low concentration. At concentrations greater than 0.2 mg/mL, the superoxide radical scavenging rates of FSI, dFSI′ and vitamin C were all over 80%, and the scavenging rates of FSI and dFSI′ were 91.6 and 83.6%, respectively (Figure S9). These results suggest that FSI showed strong scavenging activity on superoxide radicals such as vitamin C, and this scavenging activity of fucan sulfate should be correlated with its molecular weight. The concentrations inhibiting 50% radical generation (IC_50_) of FSI and dFSI′ were 65.71 and 83.72 μg/mL, respectively (Figure 7D). The IC_50_ of vitamin C on superoxide radical in this work was 49.68 μg/mL, which is in accordance with our previous report [28].

## 3. Conclusions

In this study, the structure sequence of FSI from the sea cucumber *Holothuria fuscopunctata* was revisited using a definitive bottom-up strategy. The oligosaccharides released from mild acid hydrolysis were clarified as disaccharides, trisaccharides, and tetrasaccharides, respectively, and their precise structure sequence was investigated through 1D/2D NMR and MS techniques. The glycosidic bond was α1,3 type without any α1,4 type, which is inconsistent with our previous report [15]. This different conclusion may be due to the previous report being based primarily on ^1^H–^1^H ROESY spectrum analysis of the low-molecular-weight product; in fact, the cross-peaks of anomeric proton H-1 and the H-4 of the subsequent unit could typically be observed as inter-residue cross-peaks for the proton–proton interatomic distances and related interatomic through-space connections. The tetrasaccharide (FV) (Scheme 1) was clearly observed as the most prevalent block in native FSI, and irregular structure regions were also observed as minor components. FSI was composed of the main tetrasaccharide repeating unit with a structure sequence of [-L-Fu_2S4S_-α1,3-L-Fuc_(2S)_-α1,3-L-Fuc_2S_-α1,3-L-Fuc-α1,3-]*_n_*. Moreover, the α1,3 glycosidic linkages between Fuc and Fuc_2S4S_ or Fuc_4S_ in FSI were selectively cleaved by mild acid hydrolysis. Aside from that, it was found from antioxidant activity analysis in vitro that both FSI and dFSI′ exhibited obvious scavenging activity on superoxide radicals. The results suggested that fucan sulfate from sea cucumber *H. fuscopunctata* can be considered as a potential antioxidant, and it may be potentially useful for the pharmaceutical and food industries.

## 4. Materials and Methods

### 4.1. Materials

Dried sea cucumber *H. fuscopunctata* was purchased from a local seafood market in Zhanjiang, Guangdong province. It was identified through gene comparison with the National Bioinformatics Center NCBI gene bank sequence. It was found that the partial sequence of this sea cucumber cytochrome oxidase subunit 1 gene is 99% similar to the *H. fuscopunctata* sequence. Amberlite FPA98Cl ion exchange resin was purchased from Rohm and Haas Company. Bio-Gel P6/P4 was from Bio-Rad Laboratories (Hercules, CA, USA). Sephadex G-10 and G-25 were from GE Healthcare Life Sciences (Uppsala, Sweden). Deuterium oxide (D_2_O, 99.9% Atom D) was obtained from Sigma-Aldrich (Shanghai, China). 1,1-diphenyl-2-picrylhydrazyl (DPPH) was purchased from Solarbio Technology Co., Ltd. (Beijing, China). Trifluoroacetic acid and ascorbic acid (Vc) was purchased from Aladdin Chemical Reagent Co., Ltd. (Shanghai, China). Salicylic acid, phenazine methosulphate (PMS), nitroblue tetrazolium (NBT), reduced nicotinamide adenine dinucleotide (NADH), and (2,2′-azinobis-(3-ethyl-benzothiazolin-6-sulfonic acid)) diammonium salt (ABTS) were purchased from Macklin Biochemical Technology Co., Ltd. (Shanghai, China). Potassium persulfate was purchased from Xinshenshi Chemical Technology Co., Ltd. (Wuhan, China). All other reagents were of analytical grade and obtained commercially.

### 4.2. Extraction and Purification of Native FSI

Native FSI was prepared according to the method described previously with a few modifications [8,15,29]. Briefly, 330 g of the dried body wall was treated with 1% papain for 6 h at 50 °C and subsequently treated with 0.25 M NaOH for 2 h at 45 °C. Ethanol was added to a final concentration of 40% to precipitate fucosylated glycosaminoglycan. After centrifugation (4000 rpm, 10 min) and removal of the precipitate, additional ethanol was added to the supernatant to a final concentration of 60%. The precipitate was collected by centrifugation (4000 rpm, 10 min) and dissolved in water before dialysis against water for 24 h. The retained solution was lyophilized and crude sulfated polysaccharide (12.6 g) was obtained with a yield of 3.8%. The obtained crude polysaccharide was applied to strong anion-exchange chromatography using an FPA98 column and eluted by H_2_O, 0.5 M, 1.0 M, 1.5 M, 2.0 M, and 3.0 M NaCl solution successively. The water fraction containing sulfated fucan (4.7 g) was confirmed by ^1^H NMR, and then was used in the following analysis as FSI.

### 4.3. Mild Acid Hydrolysis of FSI

#### 4.3.1. Optimization of Acid Hydrolysis Conditions of FSI

To optimize the appropriate conditions for obtaining a series of oligosaccharides, 50 mg of FSI was dissolved in 5 mL of 10 mM trifluoroacetic acid aqueous solution and maintained at 80 or 100 °C for 2 h, respectively. Then, 300 μL of the reaction solution was withdrawn at 1 and 2 h and then neutralized by adding 0.1 M NaOH. The hydrolysates were desalted through a Sephadex G-25 column and then analyzed by the HPGPC method.

#### 4.3.2. Mild Acid Hydrolysis of FSI by Trifluoroacetic Acid

For the preparation of size homogeneous oligosaccharides from the hydrolysates of FSI, FSI (1.8 g) was dissolved in 180 mL 10 mM trifluoroacetic acid aqueous solution. Then, the mixture was incubated at 80 °C for 13 h. During the hydrolysis process, 300 μL of the hydrolysates was withdrawn at every hour, and 0.1 M NaOH was added to adjust pH to neutral; then, each sample was subjected to HPGPC analysis to determine the Mw. The Mw method was carried out on an Agilent Technologies 1260 infinity II series (Agilent Co., Santa Clara, CA, USA) apparatus, equipped with refractive index detector (RID) using a Shodex OH-pak SB-804 HQ column (8 mm × 300 mm). When the Mw of the hydrolysates was approximately 3000 Da, 6 M NaOH was added to quench the reaction. Then, the low-molecular-weight product was reduced by 0.1 M NaBH_4_ at 50 °C for 1 h, concentrated and lyophilized after desalination by Sephadex G-25 to yield 1.4 g dFSI.

### 4.4. Purification of Oligosaccharides by Gel Permeation Chromatography

The oligosaccharide mixture dFSI was fractionated by GPC on a Bio-Gel P-6 or P-4 column (2 × 180 cm) eluted with 0.2 M NaCl at a flow rate of 0.15 mL/min. The fractions were monitored by the phenol-sulfuric acid method, and absorbance was measured at 482 nm with a UV spectrometer [30]. According to the fractionation curve, several fractions were further examined by HPGPC. This HPGPC method was performed on a Superdex Peptide 10/300 GL column (GE Healthcare, Uppsala, Sweden), and the sample was eluted with 0.2 M NaCl solution at a flow rate of 0.4 mL/min. Those fractions with similar Rt were combined, then desalted by a Sephadex G-10 column. Finally, the fractions corresponding to size-homogeneous oligosaccharides were pooled and freeze-dried to yield a series of oligosaccharides designated as FI, FII, FIII, FIV, FV, and FVI.

### 4.5. One-Dimensional (1D) and Two-Dimensional (2D) NMR Analysis of Oligosaccharides

For the one-dimensional and two-dimensional NMR analysis, about 3 mg of each oligosaccharide (FI–FVI) was dissolved in 1.0 mL of D_2_O (99.9%) and lyophilized three times. The final powder was dissolved in 500 μL of D_2_O (99.9%) in a 5 mm tube. The 1D NMR (^1^H/^13^C spectra) and 2D correlation spectra including ^1^H–^1^H COSY, TOCSY and ROESY, ^1^H-^13^C HSQC and HMBC spectra of FI, FII, FIII, FIV, and FVI were recorded on a Bruker AVANCE III 600 MHz spectrometer at 298 K with sufficient acquisition time. In particular, the 1D/2D NMR spectra of FV were acquired on a Bruker AVANCE III 800 MHz spectrometer (Bruker Biospin, Rheinstetten, Germany). All spectra were analyzed with MestReNova software (Mestrelab Research SL, Santiago de Compostela, Spain). The ^1^H and ^13^C chemical shifts were displayed relative to internal 3- (trimethylsilyl) propionic-2,2,3,3-d4 acid sodium salt (TSP) at 0.00 ppm.

### 4.6. Mass Spectrometry Analysis

Negative-ion ESI MS spectrometry analysis of oligosaccharides was carried out on an Accurate-Mass Q-EXACTIVE LC/MS spectrometer (Thermo Technologies, MA, USA). Each oligosaccharide was dissolved in purified water, typically at a concentration of 100 μg/mL. Mass spectra were acquired in the negative-ion mode under the following conditions: spray voltage 3800 V, capillary temperature 325 °C, auxiliary device temperature 380 °C. The mass spectra of the oligosaccharides were acquired in scan mode (*m*/*z* scan range 400–2000).

### 4.7. Antioxidant Activity Assays of FSI and dFSI′ In Vitro

Four different methods were used to estimate the antioxidant activity of the FSI and dFSI′ as described previously [31,32]. The sample concentration range was 0.025–6.4 mg/mL. These assays were carried out in 96-well plates; ascorbic acid (vitamin C) was used as a positive control and water was used as a negative control.

#### 4.7.1. DPPH Radical Scavenging Assay

For the DPPH• scavenging assay, the DPPH was a synthetic organic free radical, its ethanol solution was dark purple and had maximum absorption at 517 nm. With the free radical scavenger added, the lone pair of electrons was paired, and the dark purple DPPH was reduced to yellow DPPH-H molecules. Briefly, 50 µL of sample solution with various concentrations was mixed with DPPH• ethanolic solution (25 µL, 0.4 mmol/L) on a 96-well plate. The mixture was shaken vigorously after adding 100 μL of H_2_O and left to stand for 30 min. The absorbance of the resulting mixtures was recorded at 517 nm.

#### 4.7.2. Hydroxyl Radical Scavenging Assay

For the hydroxyl radical scavenging assay, based on the Fenton reaction process, Fe^2+^ and H_2_O_2_ were mixed to produce •OH in the reaction system. When •OH was captured by salicylic acid, a colored substance was produced with maximum absorption at 510 nm. With the substances that scavenge hydroxyl radicals added, it can compete with salicylic acid for •OH; then, the colored substance will decrease, and its absorbance will also decrease. Briefly, 50 μL of different concentrations (0.2–6.4mg/mL) of polysaccharide solution, ferrous sulfate solution (50 μL,9 mM), salicylic acid–ethanol solution (50 μL,9 mM), and H_2_O_2_ solution (50 μL,8.8 mM) was mixed on a 96-well plate. The mixture was incubated at 37 °C for 30 min, and the absorbance was measured at 510 nm using ascorbic acid as the positive control.

#### 4.7.3. Superoxide Radical Scavenging Assay

For the superoxide radical scavenging assay [28], •O_2_^−^ was generated by the NADH-PMS system, acted as a reducing agent toward NBT, and produced a water-insoluble blue formazan dye. The addition of free radical scavenger blocks the reaction process and intermediate products cannot be formed. The absorption value changes correspondingly to reflect the strength of the scavenging ability. Pipette 50 μL of the sample solutions with different concentrations (0.025–6.4 mg/mL), 50 μL of 156 μM NBT, 468 μM NADH and 60 μM PMS were added to a 96-well plate. The mixture was incubated for 5 min in a water bath kept at 25 °C and spectrophotometric measurement was recorded at 560 nm.

#### 4.7.4. ABTS Radical Scavenging Assay

For the ABTS radical scavenging assay [33], ABTS can be oxidized by potassium persulfate to generate blue-green free radical cation ABTS^+^. The compound was quite stable and had a maximum absorption at 734 nm. The antioxidant substance reacts with ABTS^+^ to make the reaction system fade. ABTS^•+^ (radical cation) solution was prepared through the reaction of 7 mM ABTS^+^ and 4.95 mM potassium persulfate, after incubation at room temperature in the dark for 12 h. When in use, the ABTS^•+^ solution was diluted with deionized water to an absorbance of 0.70 ± 0.02 at 734 nm. Then, 20µL of sample with various concentrations (0.2–6.4 mg/mL) was added to 200 μL of ABTS^•+^ solution on a 96-well plate. The mixture was incubated for 6 min at room temperature and absorbance was measured at 734 nm.

The radical scavenging activity of FSI and dFSI′ on DPPH radicals, hydroxyl radicals, superoxide radicals, and ABTS radicals was calculated according to the formula: Scavenging rate (%) = ((A_0_−A_1_)/A_0_) × 100, where A_0_ was the absorbance of blank sample and A_1_ was the absorbance value of the tested samples at corresponding wavelength. The effect of scavenging free radicals was shown by plotting the relationship between sample concentration and inhibition rate [28]. Antioxidant ability results of the samples were expressed as IC_50_ values.

## Figures and Tables

**Table 1:** ^1^H and ^13^C chemical shifts of the oligosaccharide FI and FII.

Fraction	Residue		Chemical Shift (ppm)					
			1	2	3	4	5	6
FI-a	F: L-Fuc_2S_-α1-	H	5.305 (*J*_1,2_ = 4.05)	4.463	4.039	3.915	4.199	1.247
		C	100.37	77.96	69.98	74.59	70.03	17.87
	rF: -3-L-Fuc-ol	H	3.742 (*J*_1,1′_ = 11.46)	4.055	3.769	3.707	4.189	1.227
			3.713 (*J*_1,2_ = 4.96)					
		C	65.51	74.35	80.88	76.80	68.53	21.37
FI-b	F′: L-Fuc_4S_-α1-	H	5.124 (*J*_1,2_ = 4.20)	3.870	4.006	4.638	4.279	1.290
		C	101.48	70.89	71.15	83.11	69.40	18.50
	rF′: -3-L-Fuc-ol	H	3.742 (*J*_1,1′_ = 11.46)	4.043	3.776	3.707	4.155	1.229
			3.713 (*J*_1,2_ = 4.96)					
		C	65.60	74.26	80.32	76.24	68.79	21.25
FII	I: L-Fuc_2S4S_-α1-	H	5.332 (*J*_1,1_ = 3.90)	4.472	4.160	4.706	4.333	1.304
		C	100.24	77.76	69.09	83.24	69.40	18.41
	rF: -3-L-Fuc-ol	H	3.739 (*J*_1,1′_ = 11.40)	4.053	3.784	3.713	4.184	1.227
			3.710 (*J*_1,2_ = 5.04)					
		C	65.48	74.29	81.06	76.81	68.57	21.37

**Table 2:** ^1^H and ^13^C chemical shifts of the oligosaccharide FIII and FIV.

Fraction	Residue		Chemical Shift (ppm)					
			1	2	3	4	5	6
FIII	A: L-Fuc_2S4S_-α1-	H	5.256 (*J*_1,2_ = 3.84)	4.348	4.086	4.575	4.357	1.159
		C	96.90	77.71	69.21	83.40	68.86	18.41
	B: -3-L-Fuc-α1-	H	5.008 (*J*_1,2_ = 4.08)	3.839	3.792	3.938	4.003	1.123
		C	101.44	69.07	78.55	71.50	69.90	18.05
	C: -3-L-Fuc-ol	H	3.596 (*J*_1,2_ = 5.40) (*J*_1,2_ = 7.20)	3.908	3.652	3.608	4.019	1.108
		C	65.57	74.33	80.16	76.16	69.00	21.09
FIV-a	AI: L-Fuc_4S_-α1-	H	5.011 (*J*_1,2_ = 3.90)	3.641	3.896	4.504	4.462	1.170
		C	100.37	71.17	71.53	83.52	69.41	18.37
	BI: -3-L-Fuc_2S4S_-α1-	H	5.232 (*J*_1,2_ = 3.48)	4.438	4.127	4.736	4.195	1.164
		C	100.46	75.72	75.07	81.74	69.63	18.62
	CI: -3-L-Fuc-ol	H	3.622 (*J*_1,1′_ = 11.48) (*J*_1,2_ = 4.62)	3.931	3.678	3.584	4.105	1.096
		C	65.39	74.22	81.28	76.71	68.52	21.35
FIV-b	AII: L-Fuc_2S4S_-α1-	H	5.226 (*J*_1,2_ = 3.82)	4.352	4.089	4.578	4.458	1.167
		C	97.18	77.62	69.03	83.48	69.11	18.47
	BII: -3-L-Fuc_2S_-α1-	H	5.192 (*J*_1,2_ = 4.02)	4.456	3.966	3.988	4.062	1.137
		C	100.31	74.41	75.99	71.66	69.83	17.94
	CII: -3-L-Fuc-ol	H	3.612 (*J*_1,1′_ = 11.48) (*J*_1,2_ = 4.62)	3.943	3.661	3.602	4.100	1.093
		C	65.42	74.48	81.02	76.64	68.46	21.34

**Table 3:** ^1^H and ^13^C chemical shifts of the oligosaccharide FV and FVI.

Fraction	Residue		Chemical Shift (ppm)					
			1	2	3	4	5	6
FV	A: L-Fuc_2S4S_-α1-	H	5.306 (*J*_1,2_ = 3.84)	4.378	4.129	4.596	4.353	1.188
		C	96.32	77.87	69.31	83.57	68.95	18.50
	B: -3-L-Fuc-α1-	H	4.968 (*J*_1,2_ = 4.00)	3.832	3.892	3.968	4.336	1.147
		C	98.99	69.36	78.07	70.99	69.54	18.12
	C: -3-L-Fuc_2S_ -α1-	H	5.223 (*J*_1,2_ = 3.92)	4.466	3.997	4.014	4.105	1.166
		C	100.58	75.87	75.30	71.86	69.89	18.25
	D: -3-L-Fuc-ol	H	3.660 (*J*_1, 1′_ = 11.60) 3.636 (*J*_1,2_ = 4.88)	3.954	3.699	3.620	4.115	1.133
		C	65.61	74.53	81.25	76.86	68.71	21.49
FVI	A: L-Fuc_2S4S_-α1-	H	5.245 (*J*_1,2_ = 3.90)	4.335	4.083	4.578	4.444	1.174
		C	97.21	77.65	69.16	83.58	69.01	18.47
	B: -3-L-Fuc_2S_-α1-	H	5.206 (*J*_1,2_ = 3.96)	4.427	3.999	3.980	4.344	1.133
		C	97.92	75.74	76.26	71.68	69.37	18.02
	C: -3-L-Fuc_2S_-α1-	H	5.208 (*J*_1,2_ = 3.92)	4.464	3.996	3.953	4.068	1.143
		C	100.34	75.81	76.58	71.94	69.83	17.96
	D: -3-L-Fuc-ol	H	3.633 (*J*_1,1′_ = 11.52) 3.603 (*J*_1,2_ = 7.44)	3.923	3.669	3.602	4.079	1.105
		C	65.44	74.43	80.92	76.77	68.63	21.34

**Table 4:** Electrospray ionization mass spectrometry (ESI-MS) data assignments of oligosaccharides.

Sample	Actual Peak	Charge	Negative Ion		Predicted Peak
			Structure	Formula	
FI	413.0748	1-	[M-H]^−^	C_12_H_22_NaO_12_S^−^	413.0730
FII	493.0324	1-	[M-Na]^−^	C_12_H_22_NaO_15_S_2_^−^	493.0298
	471.0502	1-	[M-2Na+H]^−^	C_12_H_23_O_15_S_2_^−^	471.0478
	391.0933	1-	[M-SO_3_-2Na+H]^−^	C_12_H_23_O_12_S^−^	391.0910
FIII	537.1515	1-	[M-SO_3_-2Na+H]^−^	C_18_H_33_O_16_S^−^	537.1489
	617.1092	1-	[M-2Na+H]^−^	C_18_H_33_O_19_S_2_^−^	617.1057
	639.0910	1-	[M-Na]^−^	C_18_H_32_NaO_19_S_2_^−^	639.0877
FIV	741.0282	1-	[M-Na]^−^	C_18_H_31_Na_2_O_22_S_3_^−^	741.0264
	719.0463	1-	[M-2Na+H]^−^	C_18_H_32_NaO_22_S_3_^−^	719.0445
	639.0890	1-	[M-SO_3_-2Na+H]^−^	C_18_H_32_NaO_19_S_2_^−^	639.0877
	617.1069	1-	[M-SO_3_-3Na+2H]^−^	C_18_H_33_O_19_S_2_^−^	617.1057
FV	432.0491	2-	[M-2Na]^2−^	C_24_H_41_NaO_26_S_3_^2−^	432.0473
	785.1463	1-	[M-SO_3_-2Na+H]^−^	C_24_H_42_NaO_23_S_2_^−^	785.1456
	887.0854	1-	[M-Na]^−^	C_24_H_41_Na_2_O_26_S_3_^−^	887.0844
FVI	1011.1652	1-	[M-H]^−^	C_24_H_39_Na_4_O_29_S_4_^−^	1011.0051
	989.1834	1-	[M-Na]^−^	C_24_H_40_Na_3_O_29_S_4_^−^	989.0231
	967.0461	1-	[M-2Na+H]^−^	C_24_H_41_Na_2_O_29_S_4_^−^	967.0412
	887.0888	1-	[M-SO_3_-2Na+H]^−^	C_24_H_41_Na_2_O_26_S_3_^−^	887.0844
	865.1067	1-	[M-SO_3_-3Na+2H]^−^	C_24_H_42_NaO_26_S_3_^−^	865.1024
	843.1247	1-	[M-SO_3_-4Na+3H]^−^	C_24_H_43_O_26_S_3_^−^	843.1205

## Data Availability

All data generated or analyzed are available from the corresponding author on request.

## Supplementary Materials

The following supporting information can be downloaded at: https://www.mdpi.com/article/10.3390/ijms23094488/s1.
