# Supplementary material for: Structure Elucidation of Fucan Sulfate from Sea Cucumber Holothuria fuscopunctata through a Bottom-Up Strategy and the Antioxidant Activity Analysis
Source: Int J Mol Sci. 2022 Apr 19;23(9):4488. doi: 10.3390/ijms23094488 (PMC9105098; doi:10.3390/ijms23094488)
Supplement: Supplementary file 1 [file ijms-23-04488-s001.zip › ijms-1666015-supplementary.pdf]

Supplementary Material

Structure elucidation of fucan sulfate from sea cucumber *Holothuria fuscopunctata* through a bottom-up strategy and the antioxidant activity analysis

Li Gao[1], Chen Xu[1], Xuelin Tao[1], Zhichuang Zuo[1], Zimo Ning[1], Linghui Wang[1], Na Gao [1,2,*], Jinhua Zhao [1,*]

[1] School of Pharmaceutical Sciences, South-Central University for Nationalities, Wuhan, 430074, China

[2] National Demonstration Center for Experimental Ethnopharmacology Education, South-Central University for Nationalities, Wuhan 430074, China

* Corresponding authors. E-mail address: gn2008.happy@163.com (Na Gao), zhao.jinhua@yahoo.com (Jinhua Zhao)

Preparation of dFSI' by peroxide depolymerization

The depolymerized FSI (dFSI') were prepared using a modified protocol based on the previous report (Shang et al., 2018). In brief, 100 mg of FSI was dissolved in 4 mL of H_2O, 3.0 mg of copper acetate and 1 mL of 30% H_2O_2 solution was added and stirred at 35℃ for 3 h. Finally, the solution was precipitated with 2 mL of saturated sodium chloride solution and ethanol with a final concentration of 80% (v/v) and collected by centrifugation (4000 × g, 15 min). Then, the precipitate was dissolved, desalted by Sephadex G-25, concentrated and lyophilized to yield depolymerized product dFSI'. The average molecular weight of dFSI' was detected on a Shodex OHpak SB-804 HQ column and calculated as 4.6 kDa.

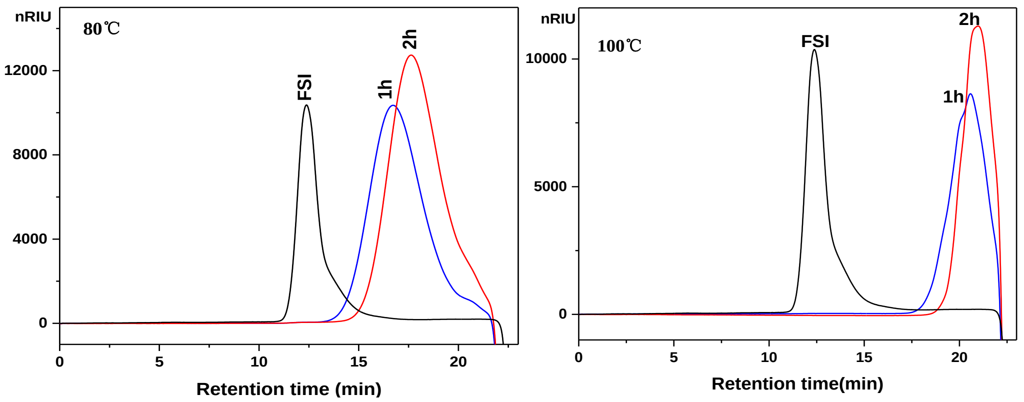

Figure S1. HPGPC profiles of acid hydrolysis hydrolysates of FSI at 80℃ and 100℃, respectively.

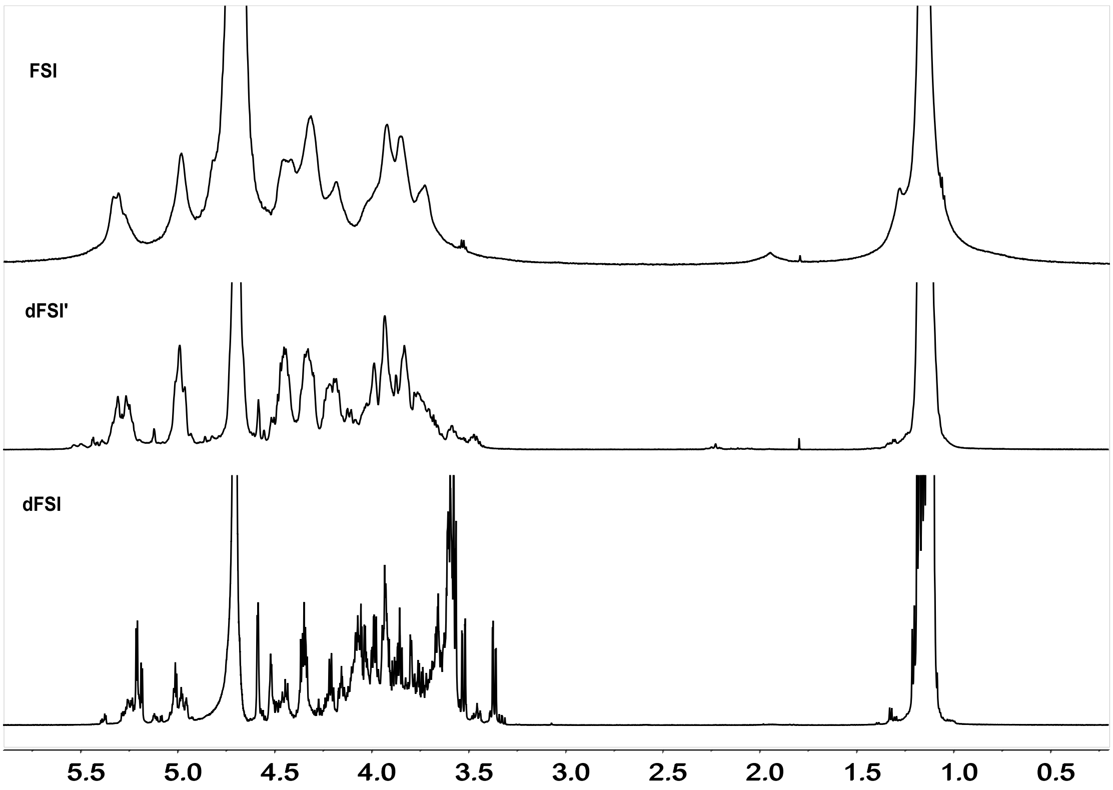

Figure S2. ^{1}H NMR spectra of native FSI from *H. fuscopunctata*, dFSI' obtained by peroxide depolymerization and dFSI obtained by mild acid hydrolysis.

A

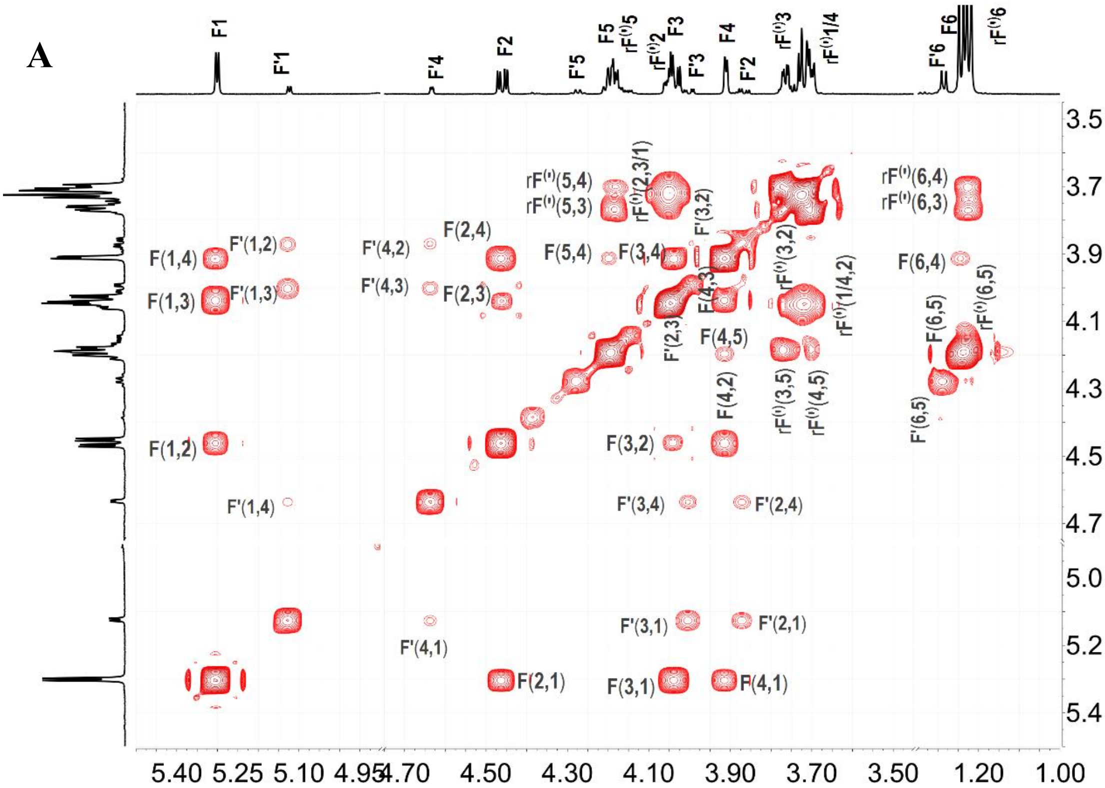
F1
F'1
F'4
F2
F'5
F5
rF(r)5
rF(r)2
F3
F'3
F4
F'2
rF(r)3
rF(r)1/4
F'6
F6
rF(r)6
F(1,4)
F(1,3)
F'(1,2)
F'(1,3)
F'(4,2)
F'(4,3)
F(2,4)
F(2,3)
F(5,4)
F(3,4)
rF(r)(5,4)
rF(r)(5,3)
rF(r)(2,3/1)
F'(3,2)
F(4,3)
F'(2,3)
F(4,5)
rF(r)(3,2)
rF(r)(1/4,2)
rF(r)(6,4)
rF(r)(6,3)
F(6,4)
F(6,5)
rF(r)(6,5)
F'(6,5)
F(4,2)
rF(r)(3,5)
rF(r)(4,5)
F(1,2)
F(3,2)
F'(1,4)
F'(3,4)
F'(2,4)
F'(4,1)
F'(3,1)
F'(2,1)
F(2,1)
F(3,1)
F(4,1)
3.5
3.7
3.9
4.1
4.3
4.5
4.7
5.0
5.2
5.4
5.40 5.25 5.10 4.95 4.70 4.50 4.30 4.10 3.90 3.70 3.50 1.20 1.00

B

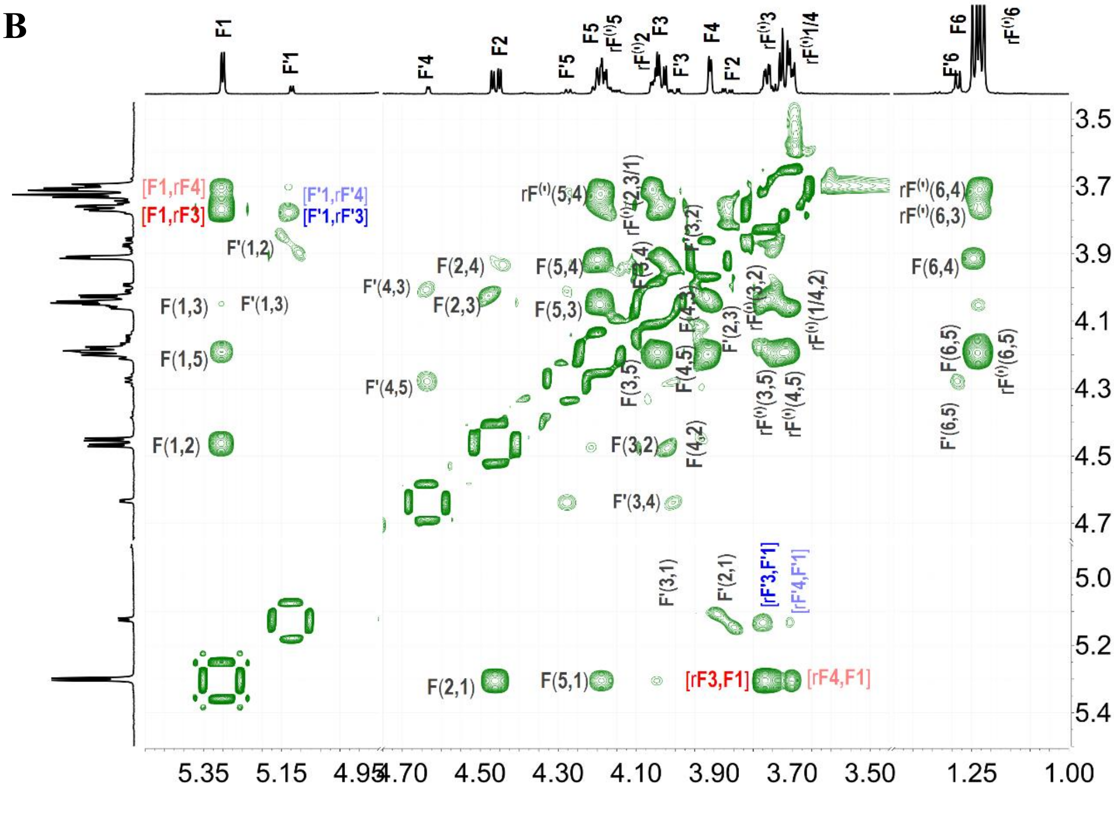
F1
F'1
F'4
F2
F'5
F5
rF(r)5
rF(r)2
F3
F'3
F4
F'2
rF(r)3
rF(r)1/4
F'6
F6
rF(r)6
[F1,rF4]
[F1,rF3]
[F'1,rF'4]
[F'1,rF'3]
F'(1,2)
F(1,3)
F'(1,3)
F(1,5)
F(1,2)
F(2,4)
F'(4,3)
F(2,3)
F'(4,5)
F(5,4)
F(5,3)
rF(r)(5,4)
rF(r)(2,3/1)
F'(3,2)
rF(r)(3,2)
rF(r)(1/4,2)
F'(2,3)
F(3,5)
F(4,5)
rF(r)(3,5)
rF(r)(4,5)
rF(r)(6,4)
rF(r)(6,3)
F(6,4)
F(6,5)
rF(r)(6,5)
F'(6,5)
F(3,2)
F(4,2)
F'(3,4)
F'(3,1)
F'(2,1)
[rF'3,F'1]
[rF'4,F'1]
F(2,1)
F(5,1)
[rF3,F1]
[rF4,F1]
3.5
3.7
3.9
4.1
4.3
4.5
4.7
5.0
5.2
5.4
5.35 5.15 4.95 4.70 4.50 4.30 4.10 3.90 3.70 3.50 1.25 1.00

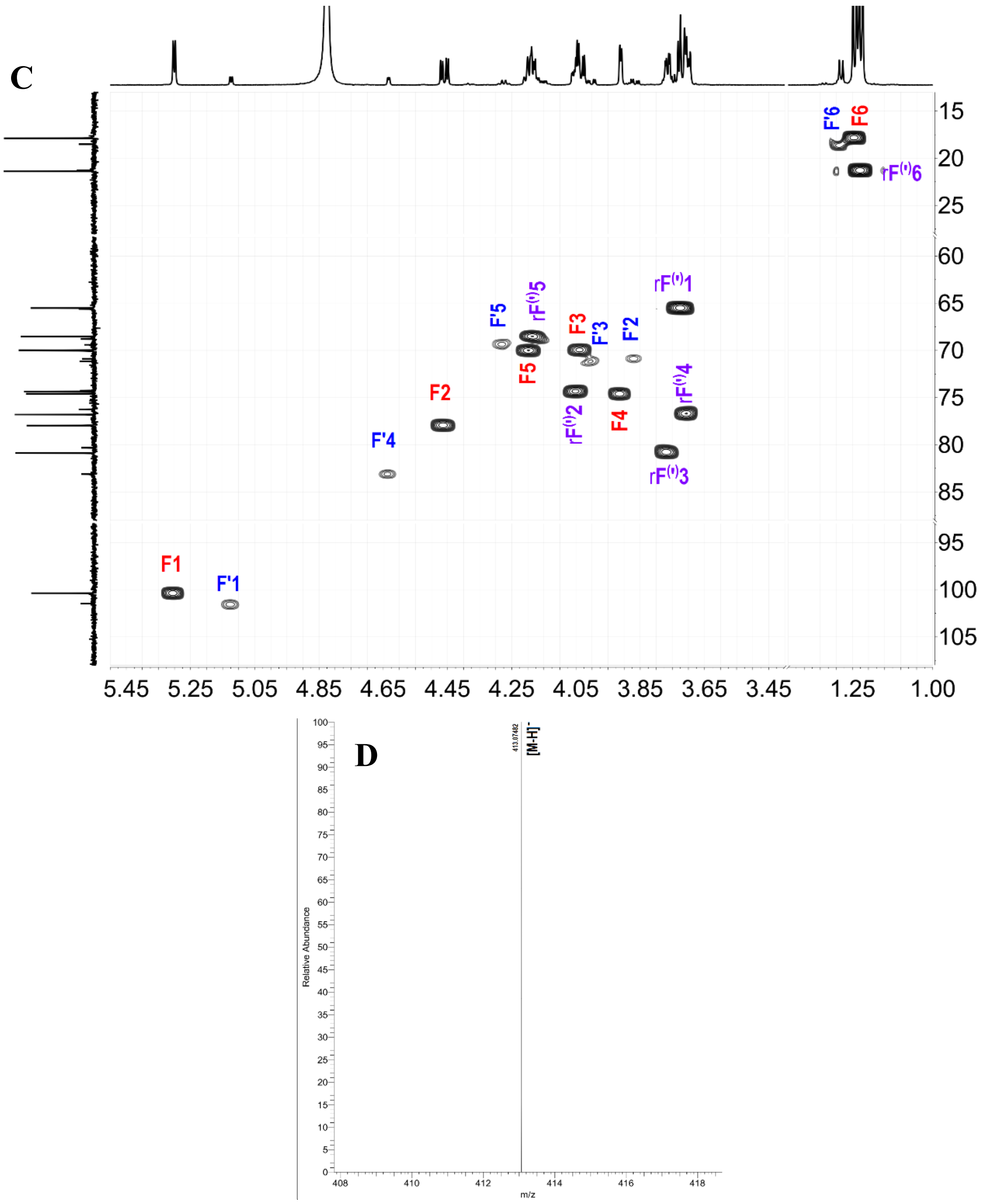

Figure S3. ^{1}H-^{1}H TOCSY (A), ^{1}H-^{1}H ROESY (B), ^{13}C-^{1}H HSQC (C) spectra of **FI** and its MS spectrum (D).

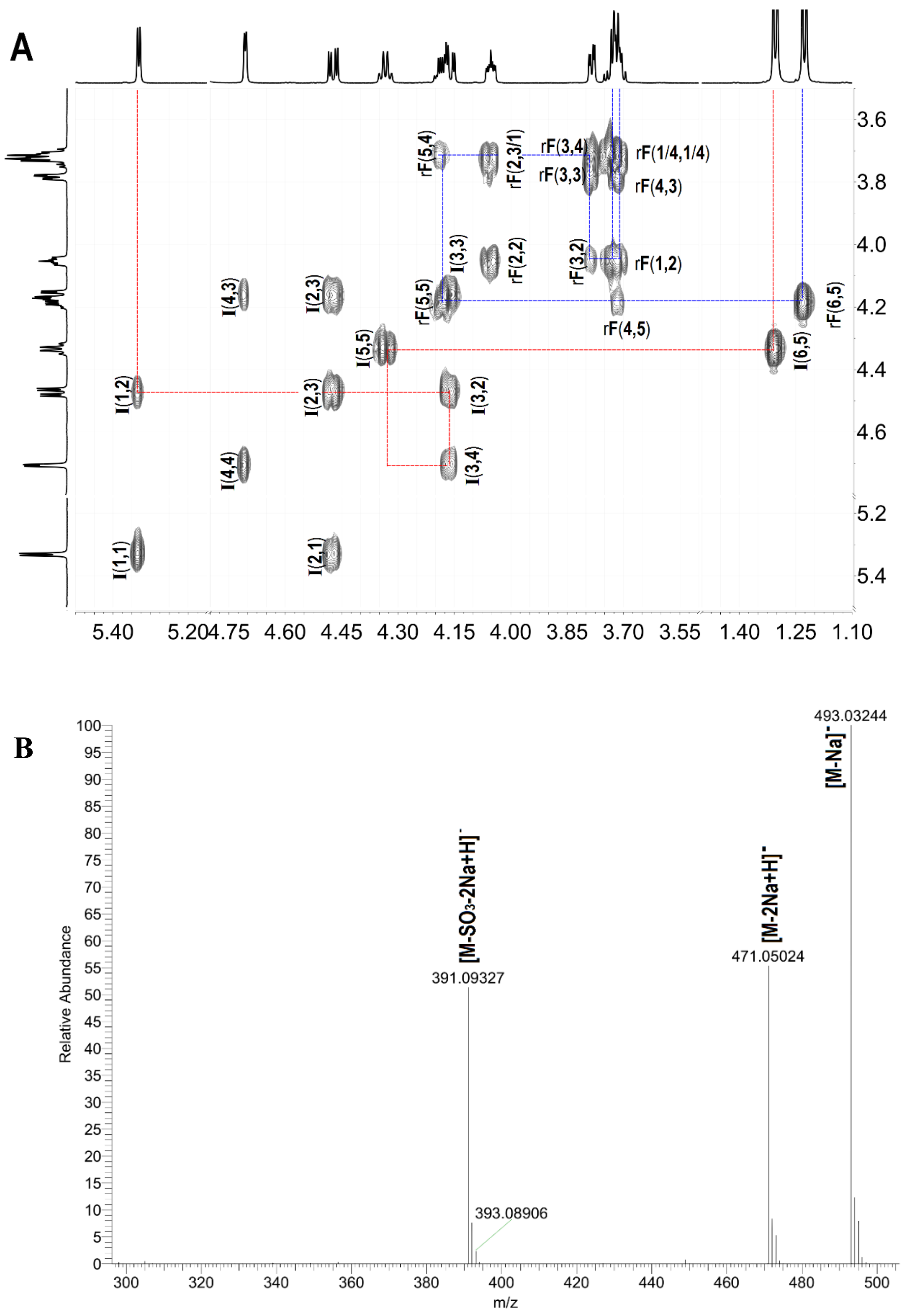

Figure S4. ^{1}H-^{1}H COSY (A) spectrum of **FII** and its MS spectrum (B).

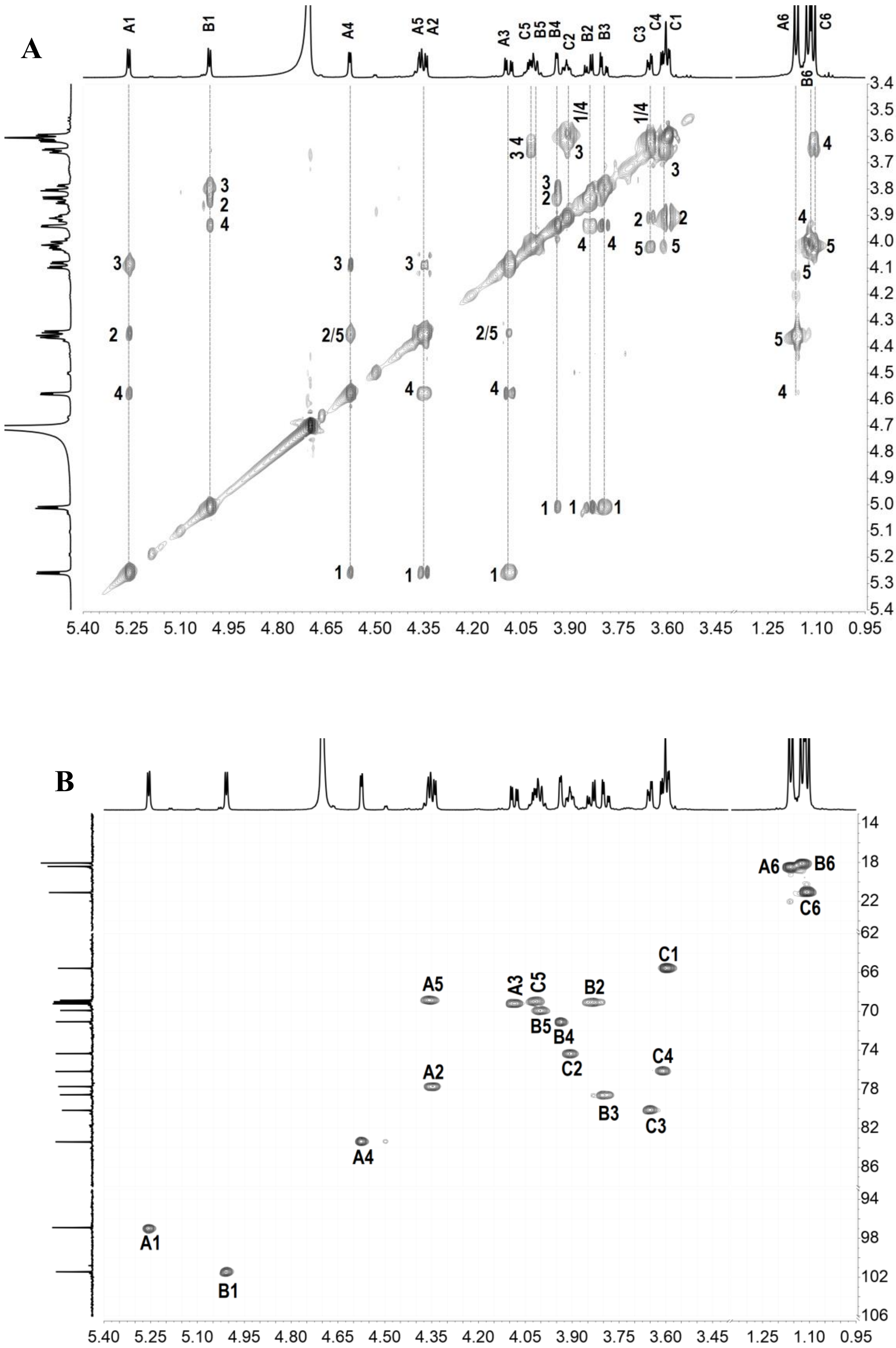
A
A1 B1 A4 A5 A2 A3 C5 B5 B4 C2 B2 B3 C3 C4 C1 A6 C6 B6
1/4
3 4
3
2
4
2/5
1
5
3.4 3.5 3.6 3.7 3.8 3.9 4.0 4.1 4.2 4.3 4.4 4.5 4.6 4.7 4.8 4.9 5.0 5.1 5.2 5.3 5.4
5.40 5.25 5.10 4.95 4.80 4.65 4.50 4.35 4.20 4.05 3.90 3.75 3.60 3.45 1.25 1.10 0.95
B
A6 B6 C6 C1 A5 A3 C5 B2 B5 B4 C2 C4 A2 B3 C3 A4 A1 B1
14 18 22 62 66 70 74 78 82 86 94 98 102 106
5.40 5.25 5.10 4.95 4.80 4.65 4.50 4.35 4.20 4.05 3.90 3.75 3.60 3.45 1.25 1.10 0.95

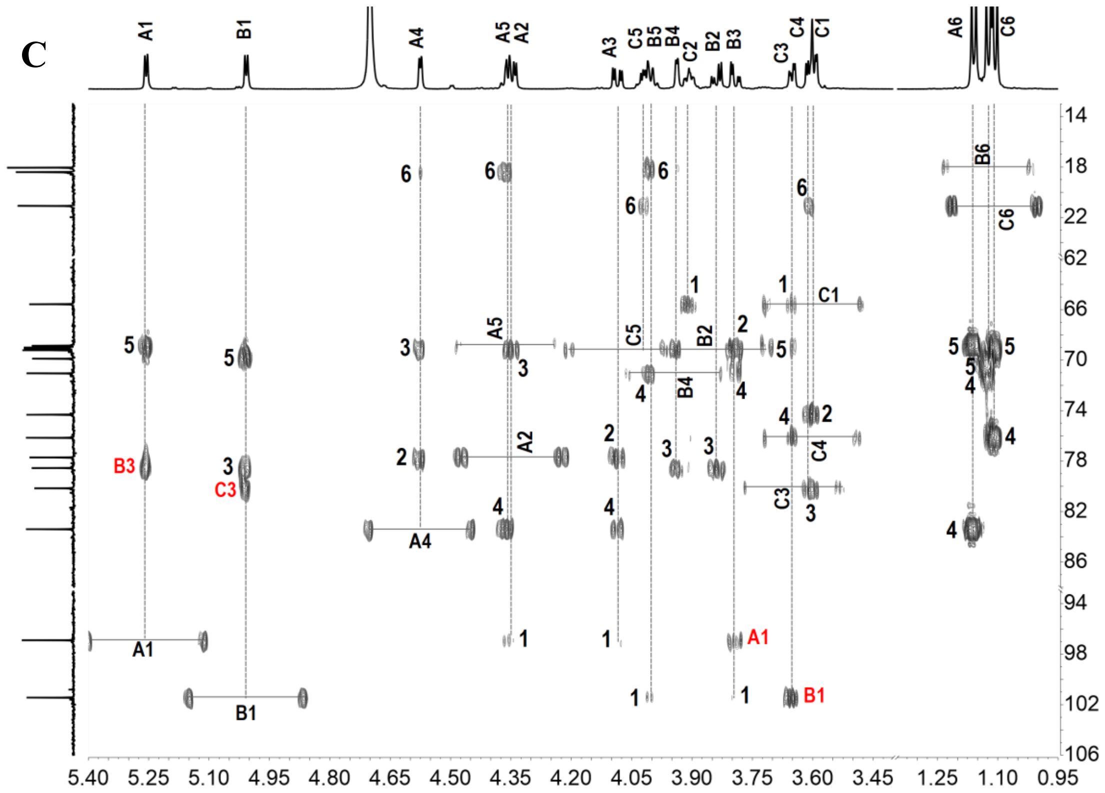

Figure S5. ^{1}H-^{1}H TOCSY (A), ^{1}H- ^{13}C HSQC (B) and ^{1}H -^{13}C HMBC (C) spectra of **FIII**.

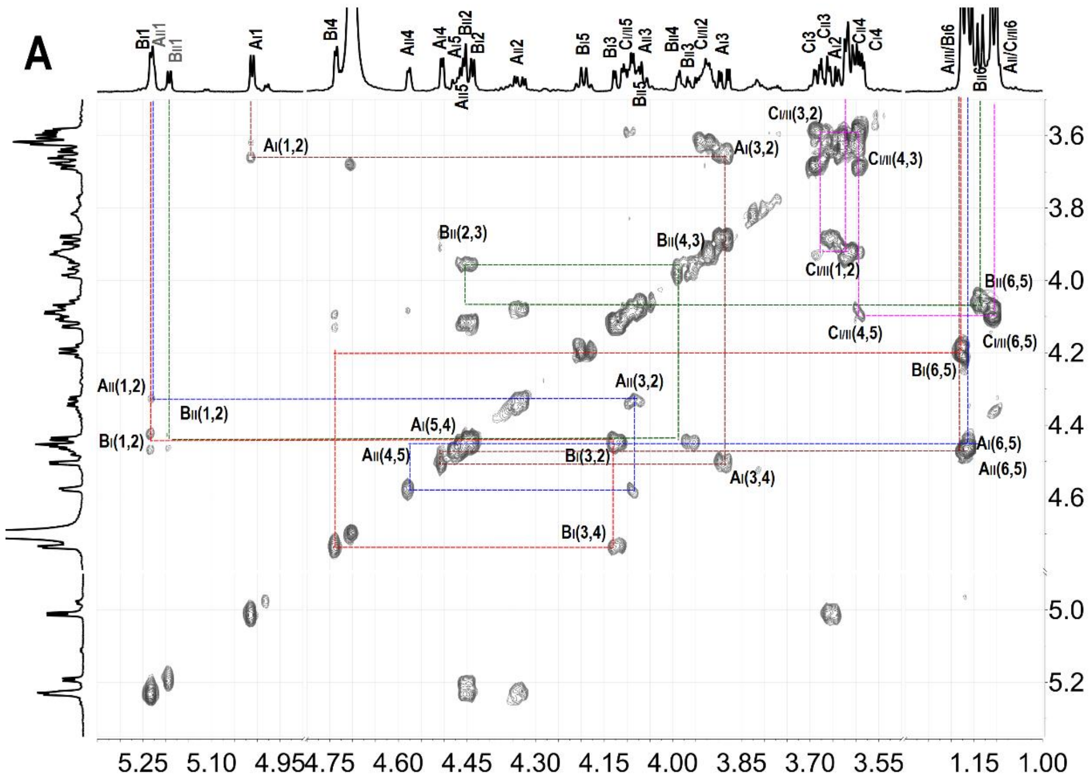

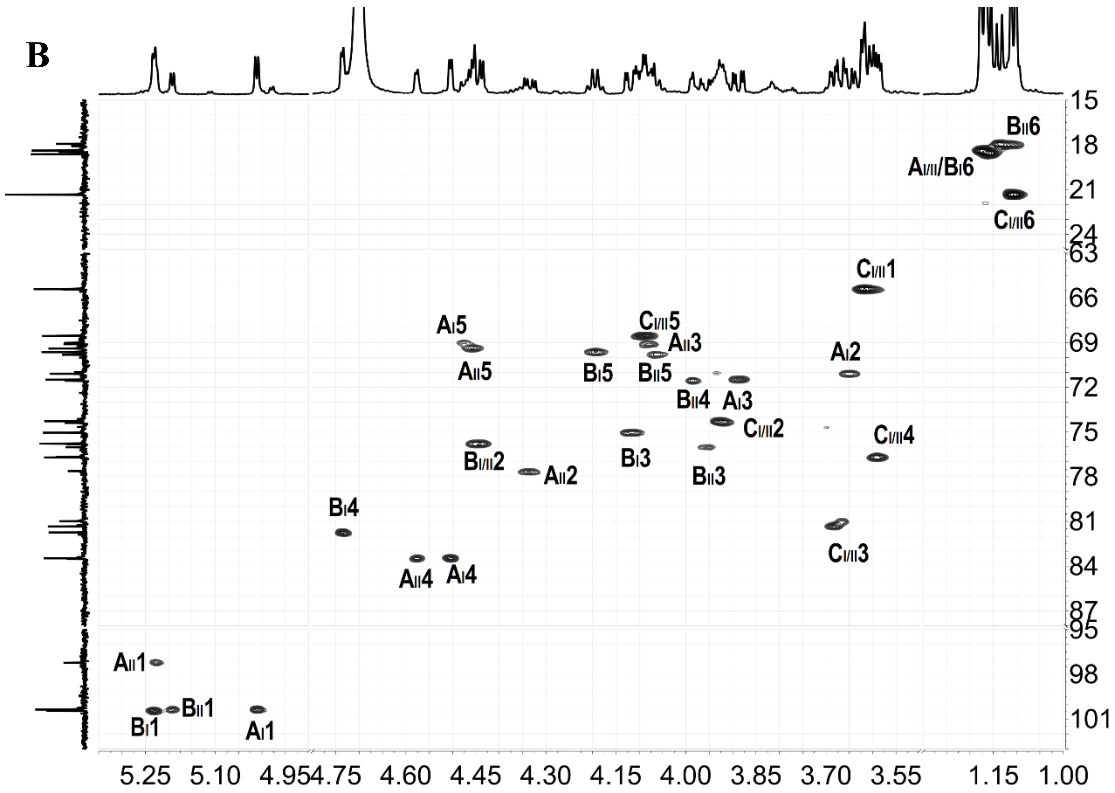

Figure S6. The ^{1}H-^{1}H COSY (A) and ^{1}H-^{13}C HSQC (B) spectra of oligosaccharides **FIV**.

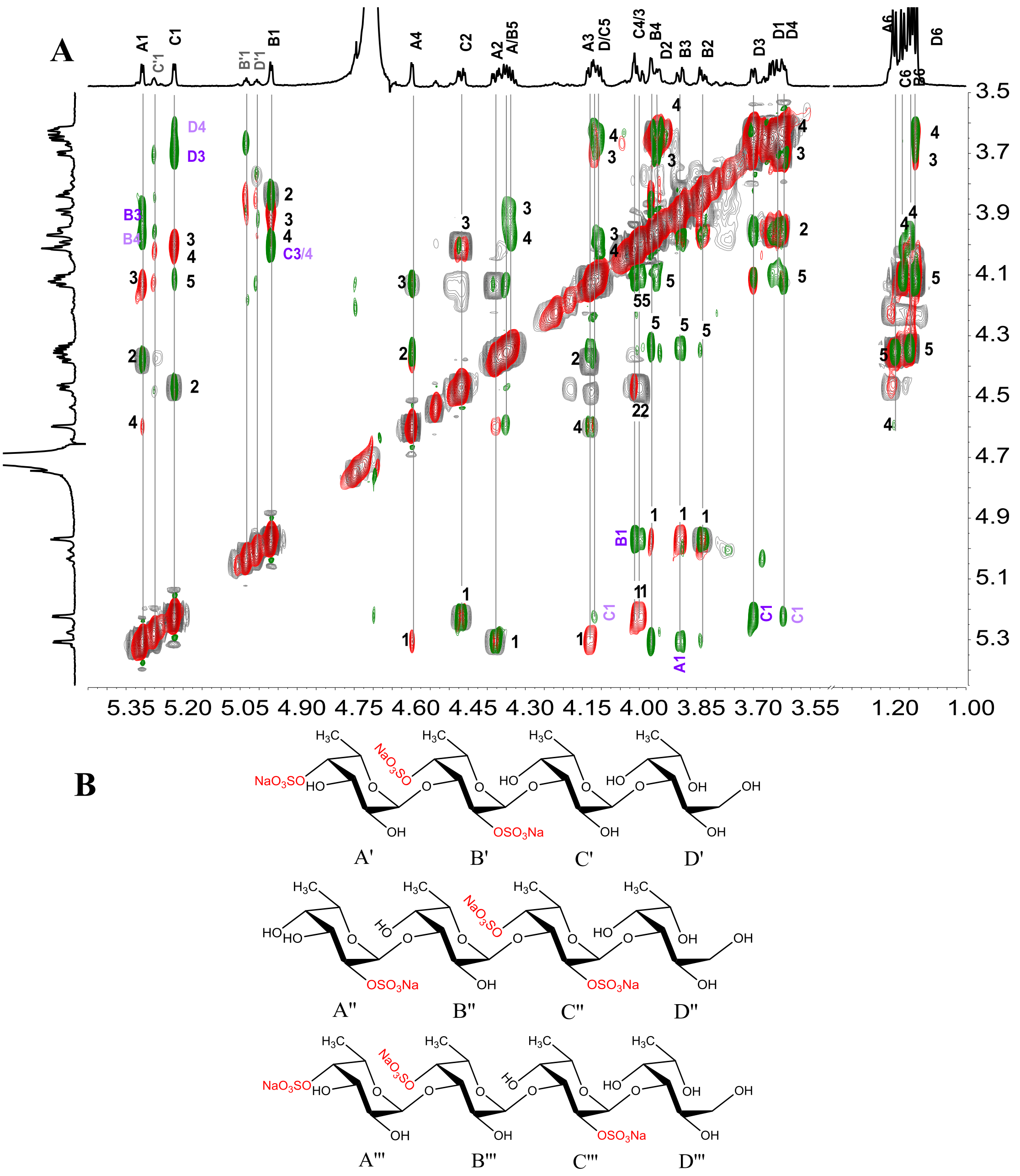

Figure S7. The overlapped spectra (A) of ^{1}H-^{1}H COSY (gray), TOCSY (red) and ROESY (green) of oligosaccharides **FV** and the structure of the minor components in **FV** (B).

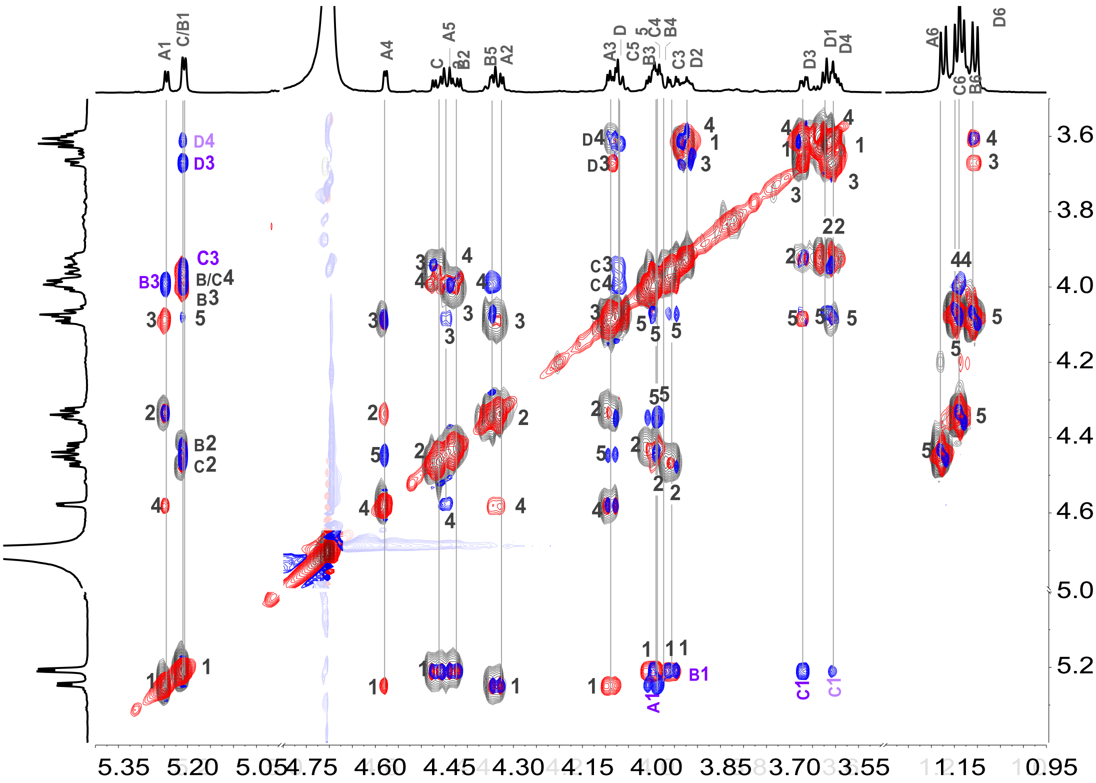

Figure S8. The overlapped spectra of ^{1}H-^{1}H COSY (gray), TOCSY (red) and ROESY (blue) of oligosaccharides **FVI**.

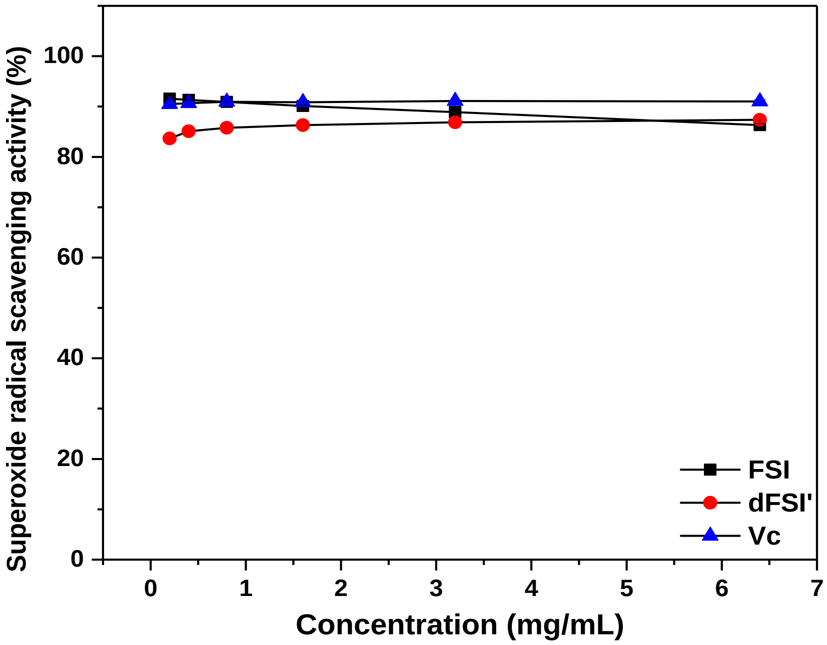

Figure S9. Scavenging effect of FSI, dFSI' and Vc on superoxide radicals at the concentration range of 0.2 - 6.4 mg/mL.

Shang, F., Mou, R., Zhang, Z., Gao, N., Lin, L., Li, Z., et al. (2018). Structural analysis and anticoagulant activities of three highly regular fucan sulfates as novel intrinsic factor Xase inhibitors. *Carbohydrate Polymers, 195*, 257-266.
